# Robust human locomotion and localization activity recognition over multisensory

**DOI:** 10.3389/fphys.2024.1344887

**Published:** 2024-02-21

**Authors:** Danyal Khan, Mohammed Alonazi, Maha Abdelhaq, Naif Al Mudawi, Asaad Algarni, Ahmad Jalal, Hui Liu

**Affiliations:** ^1^ Department of Computer Science, Air University, Islamabad, Pakistan; ^2^ Department of Information Systems, College of Computer Engineering and Sciences, Prince Sattam Bin Abdulaziz University, Al-Kharj, Saudi Arabia; ^3^ Department of Information Technology, College of Computer and Information Sciences, Princess Nourah Bint Abdulrahman University, Riyadh, Saudi Arabia; ^4^ Department of Computer Science, College of Computer Science and Information System, Najran University, Najran, Saudi Arabia; ^5^ Department of Computer Sciences, Faculty of Computing and Information Technology, Northern Border University, Rafha, Saudi Arabia; ^6^ Cognitive Systems Lab, University of Bremen, Bremen, Germany

**Keywords:** human activity recognition, smart IMU, GPS, deep learning, convolutional neural network, long short-term memory human activity recognition, GPS sensor, convolutional neural network (CNN)

## Abstract

Human activity recognition (HAR) plays a pivotal role in various domains, including healthcare, sports, robotics, and security. With the growing popularity of wearable devices, particularly Inertial Measurement Units (IMUs) and Ambient sensors, researchers and engineers have sought to take advantage of these advances to accurately and efficiently detect and classify human activities. This research paper presents an advanced methodology for human activity and localization recognition, utilizing smartphone IMU, Ambient, GPS, and Audio sensor data from two public benchmark datasets: the Opportunity dataset and the Extrasensory dataset. The Opportunity dataset was collected from 12 subjects participating in a range of daily activities, and it captures data from various body-worn and object-associated sensors. The Extrasensory dataset features data from 60 participants, including thousands of data samples from smartphone and smartwatch sensors, labeled with a wide array of human activities. Our study incorporates novel feature extraction techniques for signal, GPS, and audio sensor data. Specifically, for localization, GPS, audio, and IMU sensors are utilized, while IMU and Ambient sensors are employed for locomotion activity recognition. To achieve accurate activity classification, state-of-the-art deep learning techniques, such as convolutional neural networks (CNN) and long short-term memory (LSTM), have been explored. For indoor/outdoor activities, CNNs are applied, while LSTMs are utilized for locomotion activity recognition. The proposed system has been evaluated using the k-fold cross-validation method, achieving accuracy rates of 97% and 89% for locomotion activity over the Opportunity and Extrasensory datasets, respectively, and 96% for indoor/outdoor activity over the Extrasensory dataset. These results highlight the efficiency of our methodology in accurately detecting various human activities, showing its potential for real-world applications. Moreover, the research paper introduces a hybrid system that combines machine learning and deep learning features, enhancing activity recognition performance by leveraging the strengths of both approaches.

## 1 Introduction

The advancement of sensing technologies (Jiang and He, 2020; Zheng et al., 2023a), notably has catalyzed progress in human activity recognition (HAR). These sensors, pivotal in health (Sobhan et al., 2021; Hussain et al., 2022; Zheng et al., 2023b) and safety monitoring (Reddy et al., 2016; Mao et al., 2022a) in smart environments (Guo et al., 2022; Jiawei et al., 2022; Liu et al., 2023g), aim to be both accurate and nonintrusive. Wearable sensors (Saboor et al., 2020; Bhelkar and Shedge, 2016; Perez and Zeadally, 2021) with their potential to capture granular movement data, have introduced new possibilities in HAR (Liu et al., 2023a). However, their challenges (Saboor et al., 2020; Liu and Schultz, 2019; Bhelkar and Shedge, 2016) concerning battery life and user acceptance underscore the importance of a balanced approach. Tools like infrared sensors (Perez and Zeadally, 2021; Liu et al., 2022a) and recent 3D data acquisition systems (Yu et al., 2023; Bruno et al., 2015) such as Microsoft Kinect (Zhao et al., 2023; Liu et al., 2022b; Shen et al., 2022) are emerging as robust alternatives, offering precision without compromising user privacy. As HAR technologies evolve, integrating wearables and non-intrusive sensors, the field is poised to offer deeper insights into human behavior (Zhang et al., 2012b; Puangragsa et al., 2022) enhancing security, health monitoring, and infrastructure management (Kamarudin, et al., 2014; Hu et al., 2022; Hassan and Gutub, 2022).

This research paper discusses the application of a Smart inertial measurement unit (IMU), global positioning system (GPS), and audio sensors, along with ambient sensors, for human activity recognition (Zheng et al., 2022; Meng et al., 2022). The combination of these sensors offers a comprehensive approach to capturing diverse aspects of human movements and actions. IMUs, which consist of accelerometers, gyroscopes, and magnetometers, provide precise motion and orientation data. In conjunction with Ambient Sensors that capture contextual information, these sensors provide insight into human activities in real-world scenarios. To achieve accurate and context-aware activity recognition, advanced signal processing techniques are used to extract relevant features from the data these sensors collect. Novel feature extraction methods have been designed for signal (Hartmann et al., 2022; Hartmann et al., 2023), GPS, and audio sensor data, enriching the system’s ability to discern patterns and characteristics associated with different activities. To effectively process the information from GPS, Audio, and IMU Sensors, a Yeo-Johnson power transformation is applied for optimization. Simultaneously, IMU and Ambient features are optimized and harnessed for the identification of locomotion activities, showcasing the versatility of the proposed approach. Given the complexity and diversity of human activities, state-of-the-art deep learning techniques are employed to develop a robust and accurate HAR system (Qi et al., 2022; Wang et al., 2022; Yan et al., 2023; Ronald et al., 2021; Poulose et al., 2022; Poulose et al., 2019a). Convolutional neural networks (CNN) (Zhang et al., 2023; Wen et al., 2023a; Gangothri et al., 2023; Leone et al., 2022) are used for recognizing indoor/outdoor activities, while long short-term memory (LSTM) (Yao et al., 2023; Zheng, Y. et al., 2022) networks are chosen for locomotion activity recognition (Hu et al., 2023; Liu and Schultz, 2018; Liu et al., 2022c). The integration of CNN and LSTM allows the system to leverage spatial and temporal dependencies, thus enhancing overall recognition performance. The proposed HAR system (Zhou and Zhang, 2022; Xue and Liu, 2021; Zhao et al., 2022) is evaluated using the Opportunity and Extrasensory datasets, which are well-established benchmarks in the field of localization activity recognition (Zhu et al., 2023; Qu et al., 2023a; Qu et al., 2023b; Liu et al., 2023a). The results underscore the effectiveness of the approach, achieving remarkable accuracies of 97% and 89% for locomotion activity over the Opportunity and Extrasensory datasets, respectively, and 96% for localization activity over the Extra-sensory dataset. These findings attest to the potential of Smart IMU, GPS, Audio, and Ambient Sensors in precisely identifying and classifying a range of human activities (Gioanni et al., 2016). Beyond exploring deep learning techniques, this research paper introduces a hybrid system (She et al., 2022; Liang et al., 2018; Liu et al., 2022d; Vrskova et al., 2023; Surek et al., 2023) that blends machine learning and deep learning features. By capitalizing on the strengths of both paradigms, the hybrid system further sharpens activity recognition, signaling a promising avenue for future research and development. The primary findings and contributions of this study are outlined below:

• Development of robust denoising techniques tailored for signal and Audio sensor data, enhancing activity recognition accuracy.
• Extracting novel features for detecting human localization information.
• Development of a hybrid system that combines machine learning and deep learning features to further improve activity recognition performance.
• Furthermore, a comprehensive analysis was performed on well-known benchmark datasets, which feature diverse human actions and advanced sensors.

The subsequent sections of this paper are organized as follows:

Section 2 presents a comprehensive literature review of existing methods in the field of human activity recognition. In Section 3, the proposed system is thoroughly discussed. The experimental setup and the results obtained from the conducted experiments are outlined in Section 4. In Section 5, we discuss the system’s performance, limitations, and future directions. Finally, in Section 6 conclusions drawn from the research are presented.

## 2 Literature review

Various methods exist for recognizing human activity, with some researchers utilizing RGB cameras, others employing wearable sensors, and some leveraging multimodal sensor approaches.

### 2.1 Visual sensor-based human locomotion recognition

A new technique for pulling out details about joints and skeletons from images was introduced in a study (Batchuluun et al., 2021). The method started by changing an original thermal image, which had 1 channel, into an image with 3 channels. This change was done to combine the images in a way that would help get better results when pulling out information. The study used a tool called a generative adversarial network (GAN) to help extract details about joints and skeletons. Furthermore, the study tried to recognize different human actions using the information pulled out about joints and skeletons. The recognition of human actions was done by using two tools together: a CNN and LSTM. When they tested their method using their own collected data and also open data, the study found that their method worked well compared to other top methods. However, the system could not detect images that have low spatial textual information, due to which the system causes low performance. The study (Yin et al., 2021) developed a model to detect different human actions in a real-time healthcare environment. The authors utilized a multichannel LSTM. This system, built to detect actions through three-dimensional skeleton data, incorporated a unique loss function to enhance its accuracy. They used two benchmark datasets: one is NTU RGB + D and the second is TST fall detection datasets. However, the system has limitations in achieving flawless skeleton data due to a frame-level error detection approach and struggles with identifying the roots of issues related to dimensionality, which in turn impacts the overall accuracy of the system. In another study (Chen et al., 2023), the authors concentrated on recognizing actions through different video frames. Residual CNN and a second spatial attention module are utilized for the recognition of actions. The proposed system does not have integrated optical flow maps, which adversely impacts the performance of the system.

### 2.2 Human locomotion recognition via wearable technology

In the work conducted by Mutegeki and Han, (2020), an integrative deep learning architecture for activity recognition was introduced, utilizing a CNN-LSTM model. This approach aimed to enhance predictive accuracy for human activities derived from raw data while simultaneously reducing model complexity and negating the necessity for intricate feature engineering. The pro-posed CNN-LSTM network was devised to be deep in both spatial and temporal dimensions. The model manifested a 99% accuracy rate on the iSPL dataset (an internal dataset) and 92% on the publicly available UCI HAR dataset. However, the findings indicate a decline in performance when addressing complex actions, such as atomic-level activities. Additionally, as the model complexity amplified, the SoftMax loss also escalated, suggesting that the concurrent use of CNN and LSTM layers did not enhance the outcomes. Jaramillo et al. (2022) utilized a technique called Quaternion filtration by using single sensor data. In the next step, different segmentation techniques have been used to segment the data. Subsequently, features are extracted. Finally, for the classification of activities, the LSTM classifier has been utilized. We identified that the system is more computationally expensive. Hu et al. (2023), presents a system for human activity recognition is presented using IMU sensors, and the data was collected from Wearable devices. Different techniques are utilized to preprocess the data, including moving averages, sliding overlap windows, and data segmentation. For recognition of activities, five different classifiers are used including CNN, recurrent neural network, LSTM, bidirectional LSTM (BiLSTM), and gate recurrent unit. Due to a huge number of epochs, the proposed system is very expensive in terms of time complexity. Recently, the hidden Markov model (HMM) has entered the field of vision of researchers (Liu and Schultz, 2018). Its inherently logical modeling capability of time series endows human activity recognition with a certain degree of interpretability.

### 2.3 Human locomotion recognition through multisensor systems

The study (Hanif et al., 2022) presents a multimodal locomotion system, utilizing the Opportunity++ and HWU-USP datasets for their study. The data was subjected to various pre-processing techniques; for image-based data, the skeleton was initially extracted, while for inertial sensors, the noise was removed followed by segmentation. Various features, including Pearson correlation, linear prediction, and cepstral coefficients, were extracted. The classification of locomotion was performed using a recursive neural network. Nonetheless, the confidence levels obtained for each extracted skeleton body point do not meet the desired standards, particularly for both ankle points. In another multimodal system, proposed (Nafea et al., 2022) data was collected using smart devices. For preprocessing the raw sensor data, different methods such as filtration, windowing, and segmentation were utilized. Multiple features were extracted, including time-based, statistical, frequency-based, and rotational features. Furthermore, various machine learning classifiers have been explored to classify both complex and basic activities, such as k nearest neighbour ( k -NN), neural networks, and Naïve Bayes. However, these learning approaches tend to be susceptible to errors and often deliver suboptimal accuracy in the context of human locomotion recognition (HLR), resulting in performance that does not achieve satisfactory outcomes. In another study (Ma et al., 2023) a system was proposed to remotely monitor people, utilizing multimodal sensors to monitor activities. CNN and gated recurrent unit (GRU) were explored for recognizing different human activity patterns. Nonetheless, the suggested approach did not yield strong results due to significant losses in both the training and validation sets (M. Ronald. et al., 2021). use the iSPLInception model a deep learning architecture based on the synergistic combination of Inception modules and ResNet strategies. By refining these components, the model achieves a significant balance between depth and computational efficiency, essential for real-time processing. The researchers focused on enhancing predictive accuracy for HAR while ensuring the model’s feasibility on devices with constrained computational resources. Through extensive benchmarking across diverse datasets, the iSPLInception demonstrates robustness in classifying a variety of activities. A comparison with other deep learning models such as LSTMs and CNNs confirmed its superior performance, making a notable contribution to the HAR domain. The methodology outlined by the authors provides a scalable solution that paves the way for future research in activity recognition using wearable sensor data. Poulose. et al. (2022) proposes an innovative approach to human activity recognition (HAR) using a system referred to as HIT (Human Image Threshing) machine. This system employs a smartphone camera to capture activity videos, which are then processed using a mask region-based convolutional neural network (R-CNN) for human body detection. The process also includes a facial image threshing machine (FIT) for image cropping and resizing. The core of the HIT machine’s methodology is its ability to clean and preprocess data, followed by deep feature extraction and model building for activity classification. The system is tested with various deep learning models like VGG, Inception, ResNet, and EfficientNet, achieving remarkable accuracy in classifying activities such as sitting, standing, walking, push-ups, dancing, sit-ups, running, and jumping. This approach significantly outperforms traditional sensor-based HAR systems, demonstrating the effectiveness of vision-based activity recognition using deep learning models.

## 3 Materials and methods

### 3.1 System methodology

**FIGURE 1:**
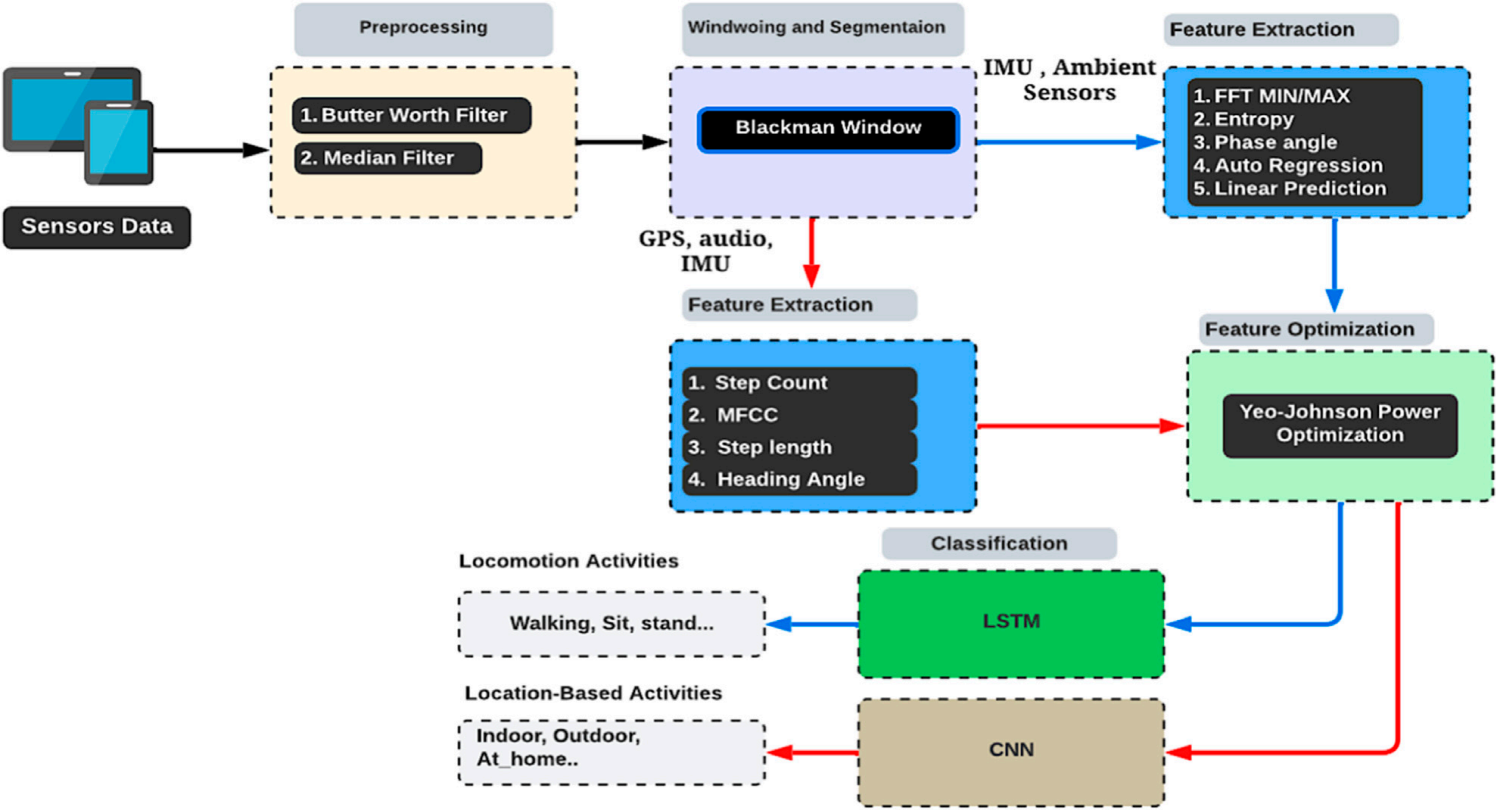
The architecture of the proposed system.

In this work, we follow a multistep approach to process and analyze data from different types of sensors (Ahmad, 2022; Zhang et al., 2022a; Latha et al., 2022). Initially, we address the issue of noise in the raw signal and use distinct filters for each sensor type. Specifically, we use a Butterworth filter for the IMU and Ambient sensors and a median filter for GPS and audio data. Next, to efficiently handle large sequence data, we utilize windowing and segmentation techniques. This allows us to break down the data into smaller segments, facilitating more effective processing. In the third step, we focus on extracting advanced features from different types of sensors. These features include statistical, phase angle, autoregressive modelling, and linear prediction features. Additionally, for the IMU and audio data, we extract various features such as step count, step length, and Mel-frequency cepstral coefficients (MFCCs). All of these features are further optimized and combined using the Yeo-Johnson power transformation. Optimized GPS, IMU, and audio sensor features are then sent to a CNN for localization activity analysis, while the IMU and ambient sensor features are directed to an LSTM network for locomotion activity recognition (Jaiwei et al., 2022; Zhang et al., 2022b; Rustam et al., 2020). The proposed system’s architecture is visually represented in Figure 1.

### 3.2 Noise removal

The data was collected from raw sensors that include noise. Noise is unwanted data or irrelevant data due to many reasons during data collection. So, to handle the noise, we used 2 types of filters because of different types of sensor data. To remove noise from the IMU and ambient sensors, we used a third-order Butterworth filter (Bae et al., 2020; Liu et al., 2023f; Cömert et al., 2018; Sulistyaningsih et al., 2018) (i.e., n=3 ) was used. The choice of this order strikes a balance between achieving a reasonable roll-off and minimizing signal distortion. The critical frequency $f_{c}$ , was set to 10% of the Nyquist frequency, represented as $W_{n}=0.1$ . This ensures that frequencies beyond 10% of the Nyquist frequency are attenuated, providing a smooth output while preserving the essential characteristics of the input signal. The magnitude response of a Butterworth filter in the frequency domain is given by

$$\left|H\left(f\right)\right|=\frac{1}{\sqrt{1+\left({\frac{f}{f_{c}}}^{2n}\right)}}$$

$H\left(f\right)$ represents the magnitude of the filter response at frequency f . $f_{c}$ is the critical frequency, which is the frequency at which the filter’s response is $\frac{1}{\sqrt{2}}$ of its maximum (or passband) response, n denotes the order of the filter, dictating the steepness of the roll-off. Higher order results in a sharper transition between the passband and the stopband. Similarly, for the GPS and microphone sensors, we used a median filter (Altun and Barshan, 2010). To apply the median filter, we used a kernel of size 3, which essentially means that for each data point, the filter considered it and one neighboring data point on each side. The median value of these three points then replaced the original data point. Mathematically, for each component, the median of the current value and its neighbors was computed, producing the filtered data. Mathematically, the filtered acceleration for each component can be expressed as

$$S_{x}=\text{median}(x\left[i-k\right],x\left[i-k+1\right],\ldots .x\left[i+k\right]$$

$$S_{y}=\text{median}(y\left[i-k\right],y\left[i-k+1\right],\ldots .y\left[i+k\right]$$

$$S_{z}=\text{median}(z\left[i-k\right],z\left[i-k+1\right],\ldots .z\left[i+k\right]$$

where $S_{x}$ , $S_{y}$ , and $S_{z}$ are the signal.

Post the filtering process, to synthesize a unified representation of the signal component, we then employed the Pythagorean theorem:

$${\text{magnitude}}_{\text{filtered}}=\sqrt{{\left(S_{x}\right)}^{2}+{\left(S_{y}\right)}^{2}+{\left(S_{z}\right)}^{2}}$$

**FIGURE 2:**
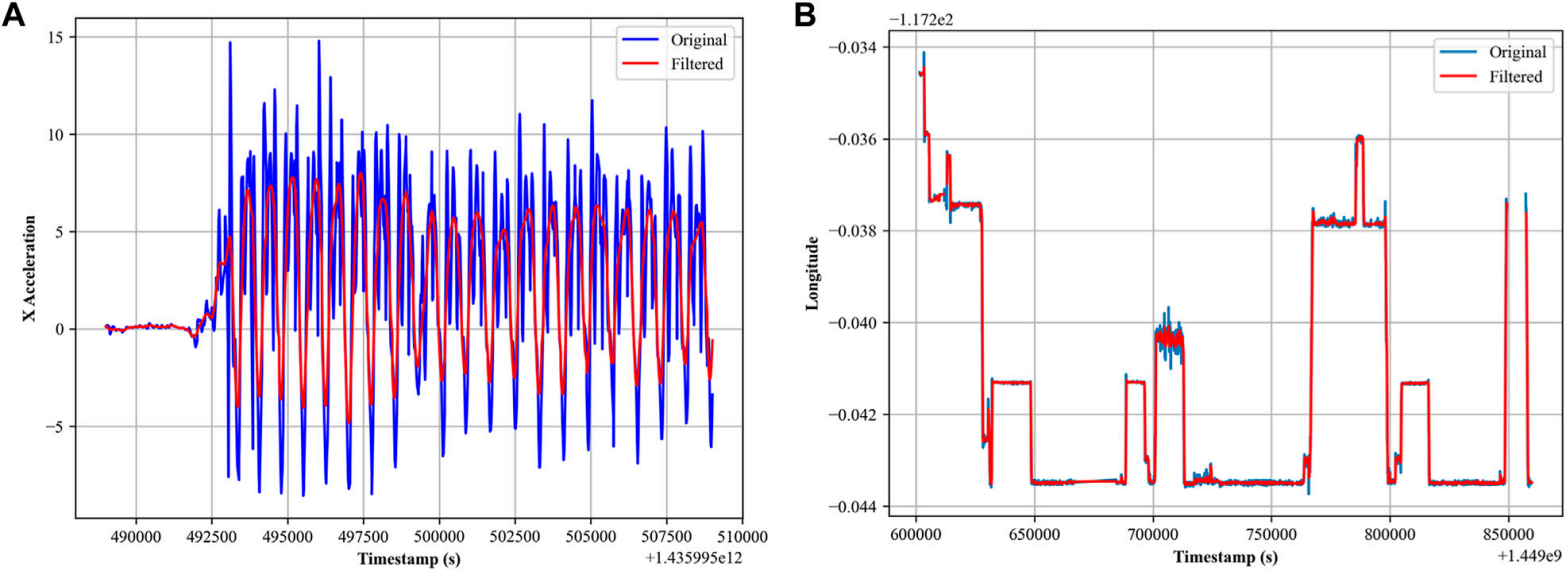
**(A)** Butterworth filter for accelerometer sensor; **(B)** median filter for GPS sensor.

However, it is important to note that the GPS sensor has less noise compared to other sensors, which can be seen in Figure 2B.

### 3.3 Windowing and segmentation

**FIGURE 3:**
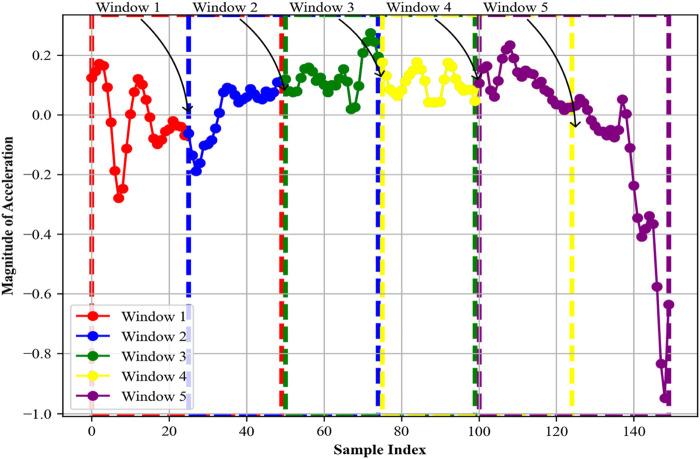
Blackman windows for the first five segments.

To window and segment large sequence data for efficient processing, we turned to the Blackman window (Kwapisz et al., 2011) windows technique to modulate the signal. Windows plays an important role in this phase. By applying a Blackman window to the signals during segmentation, we smooth the abrupt beginnings and endings of segments, thereby reducing spectral leakage, a phenomenon where energy from one frequency leaks into another, potentially obscuring important features. This ensures that the Fourier transform of the windowed signal provides a more faithful representation of its frequency content. Furthermore, in human activity recognition, activities can span varying durations and might be best represented by capturing their essence within specific windows (Poulose et al., 2019b). The Blackman window, with its inherent properties, ensures that each segmented frame is appropriately weighted, reducing discontinuities at the boundaries. This results in improved frequency domain representations, enabling more accurate feature extraction, and consequently more precise activity recognition. Mathematics of the Blackman Window is

$$W_{n}=0.42-0.5\cos\left(\frac{2\pi n}{N-1}\right)+0.8\cos\left(\frac{4\pi n}{N-1}\right)$$

where W $\left(n\right)$ is the window function. N is the total number of points in the window, and n ranges from 0 to N−1 . For our specific implementation, we used a 50-sample window to represent 5 s (He and Jin, 2008; Hao, 2021; Liu et al., 2021; Hatamikia et al., 2014) of activity with 25% overlap. After generating the Blackman window values based on the formula, we multiplied each point in our data segments with its corresponding Blackman window value. To bring clarity to our process, we visualized the results through distinct line plots, with each of the five windows represented in a unique color in Figure 3, and Algorithm 1 shows the working of the Blackman windowing technique.

Algorithm 1 Blackman Windowing and Segmentation

**Input:** Time-series data array D
   Window size *N*
**Output:** List *F* containing feature vectors for each segment
**Method:** Create a Blackman window *W* of size *N*
   Initialize an empty list *F* to store feature vectors for each segment
**For** *i* = 0 to length of *D − N* with a step size of *N:*
   Extract a segment *S from D [ i: i + N ]*
   *Multiply S with W element-wise to get* $S_{windowed}$ *​*
   Compute features f from $S_{windowed}$ *Append f to F.*
**return** list *F* containing feature vectors for each segment

### 3.4 Feature extraction for locomotion activity

Another essential step in this research is the extraction of features, ensuring that the model effectively recognizes data patterns. We derived unique features for various sensor types. For both IMU and Ambient sensors, we extracted features such as phase angle, linear predictions, FFT Max/Min, Shannon entropy, skewness, kurtosis, and autoregressive analysis.

#### 3.4.1 Phase angle

**FIGURE 4:**
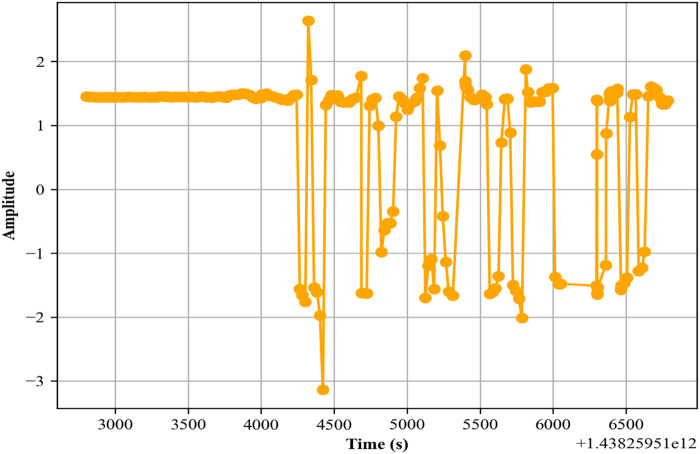
Phase angles were calculated from the accelerometer data over the Opportunity dataset.

Phase angles hold significance in signal analysis, particularly in the field of human activity recognition. Phase angles capture the temporal alignment and synchronization of cyclic movements, helping in the extraction of valuable information from complex signals (Zhang, 2012; Liu et al., 2022a). These angles provide insight into the relative timing of movements in different dimensions, enabling the identification of specific activities and patterns. Mathematically, the phase angle between two signals A and B can be calculated using the arctangent function, which takes into account the ratio of their spectral components in the frequency domain. For accelerometer data, the phase angle between the x and y components ( $\phi_{xy}$ ), x and z components ( $\phi_{xz}$ ), and y and z components ( $\phi_{yz}$ ), can be computed as

$$\phi_{xy}=\arctan\left(\frac{\text{FFT}\left(Ay\right)}{\text{FFT}\left(A_{X}\right)}\right)$$

$$\phi_{xz}=\arctan\left(\frac{\text{FFT}\left(Az\right)}{\text{FFT}\left(A_{x}\right)}\right)$$

$$\phi_{yz}=\arctan\left(\frac{\text{FFT}\left(Az\right)}{\text{FFT}\left(A_{y}\right)}\right)$$

where $\text{FFT}A_{x}$ , $\text{FFT}A_{y}$ , and $\text{FFT}A_{z}$ represent the fast Fourier transforms of the x , y , and z components of the sensor data, respectively. Figure 4 exemplifies the phase angles calculated in XY graphically.

#### 3.4.2 Auto regressive model

Autoregressive (AR) modeling (Li et al., 2020; Xu et al., 2016; Gil-Martin et al., 2020a) is a powerful technique in signal analysis, particularly for human activity recognition. It involves predicting a data point in a time series based on previous data points and capturing temporal dependencies and patterns. This is especially useful in recognizing periodic or rhythmic activities, as the model captures the repeating patterns inherent in activities like walking, running, or cycling. By comparing the predicted and actual values, deviations can be detected, helping to identify anomalies or changes in activity patterns (Bennasar et al., 2022; Liu et al., 2021). For example, variations in step lengths, gait irregularities, or sudden changes in motion can be indicative of different activities or health conditions (Wen et al., 2023). We used an AR model to model the time series data for the walking activity opportunity dataset. In an AR model, the value at time *t* is predicted as a linear combination of the *p* previous values. For an AR model of order *p,* the value $X_{t}$ at time *t* is modeled as

$$X_{t}=c+\sum_{i=1}^{p}\phi_{i}X_{t-1}+\varepsilon_{t}$$

where: $X_{t}$ , is the value at time *t, c* is a constant, $\phi_{i}$ are the parameters of the model and $\varepsilon_{t}$ is the white noise. After fitting the AR model to the data, we used the model to make predictions for future points. The prediction step is based on the AR model equation. For each future point $X_{t}$ , the predicted value is calculated as

$$\hat{X_{t}}=c+\sum_{i=1}^{p}\phi_{i}X_{t-1}$$

**FIGURE 5:**
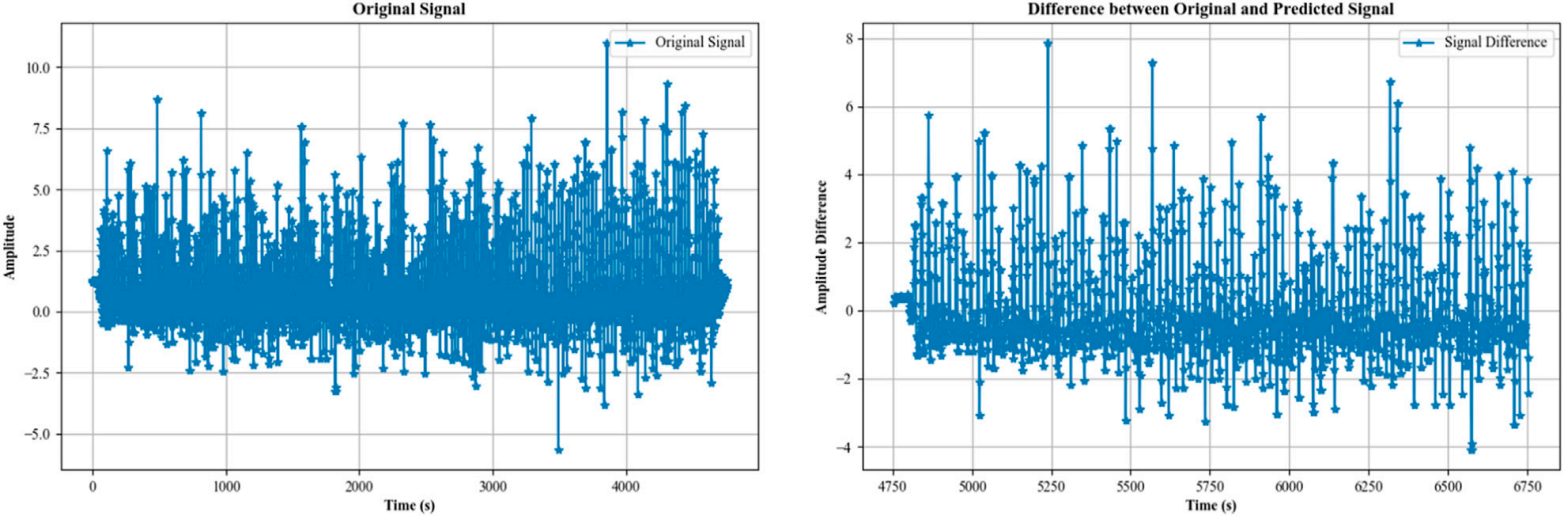
Difference between the original and predicted time series from the accelerometer data of the activity “walking” over the Opportunity dataset.

The difference between the actual AR model and the prediction step is that the actual AR model includes a noise term $\varepsilon_{t}$ while the prediction step does not. The noise term represents uncertainty and random fluctuations that cannot be predicted by the AR model. Thus, it is not included in the prediction step. Finally, we plotted the difference between original and predicted time series data in Figure 5.

#### 3.4.3 Linear prediction for signal

**FIGURE 6:**
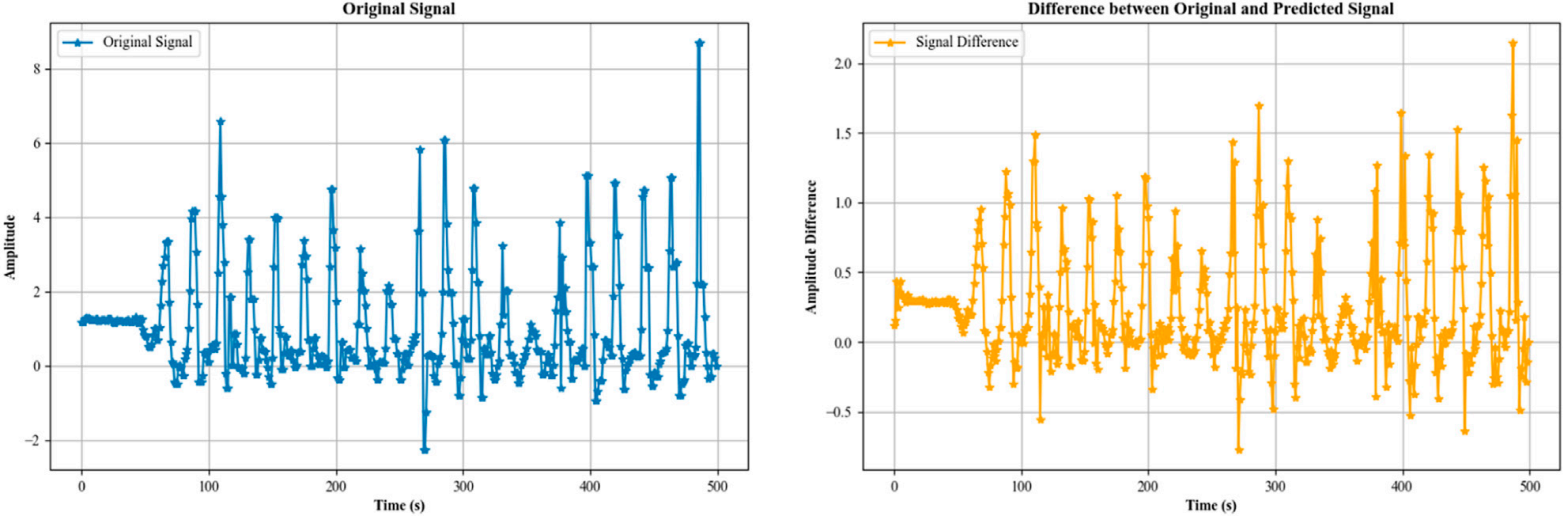
Difference between the original and linear predicted time series from the accelerometer data of the activity “walking” over the Opportunity dataset.

After calculating the autoregression, we then calculated the linear prediction. Linear prediction is a powerful method employed in signal analysis for uncovering meaningful patterns and trends in data. This approach is particularly useful when dealing with time-series data, such as movement patterns. This concept finds the relationship between current and previous data points; linear prediction enables us to forecast how the signal might evolve over time. This predictive capability enables the identification of distinctive movement patterns and characteristics that are indicative of specific activities. We preprocess the accelerometer data to ensure its quality and reliability. We then apply linear prediction techniques to model the temporal patterns of each activity. This involves training linear models that predict future data points based on a history of previous observations. The optimization of model coefficients is carried out to minimize prediction errors, resulting in predictive models that capture the underlying motion dynamics. For a time, series x_t, linear prediction estimates $x_{t}$ ​ as a weighted sum of *p* previous values $x_{t-1}$ , $x_{t-2}$ ,…… $a_{p}x_{t-p}$ :

$$x_{t=}c+a_{1}x_{t-1}+a_{1}x_{t-2}{+\cdots +a}_{p}x_{t-p}$$

where *c* is a constant term and $a_{1}$ , ${\;a}_{2}$ ,…., $a_{p}$ ​ are the coefficients of the linear model. These coefficients are determined through optimization methods that minimize the prediction error. Figure 6 portrays the linear prediction for walking activity.

#### 3.4.4 Fast fourier transformation (FFT) min/max and entropy

We first calculated FFT (Javeed et al., 2021; Li et al., 2018), a mathematical algorithm that unveils the frequency-domain representation of a time-domain signal. By applying FFT to sensor data, it becomes possible to uncover the underlying frequency components inherent in various human activities. Peaks and patterns in the resulting frequency spectrum can be associated with specific motions or actions, offering crucial insights into the dynamic nature of movements (Liu, 2021). We calculated the minimum and maximum components from the FFT spectrum. It can be calculated as

$$X\left(\;f\right)=\int_{-\infty }^{\infty }x\left(t\right)e^{-j2\pi ft}dt$$

where $X\left(\;f\right)$ , is the frequency-domain representation, $x\left(t\right)$ , is the time-domain signal, f is the frequency, and *j* is the imaginary unit. Furthermore, we extracted the Shannon entropy feature. In the context of signal analysis for human activity recognition, Shannon entropy (Khairy, 2022) can reveal the complexity and diversity of frequency components in the signal. Higher entropy values suggest a broader range of frequencies and more varied motion patterns. Mathematically, it can be computed as

$$H=-\sum_{i=1}^{N}p\left(f_{i}\right)\log2p\left(f_{i}\right)$$

where *N* is the number of frequency bins, $f_{i}$ is the *i*th frequency bin and $p\left(f_{i}\right)$ is the probability of occurrence of $f_{i}$ in the signal’s frequency distribution These features are demonstrated in Supplementary Figure S1.

#### 3.4.5 Skewness

**FIGURE 7:**
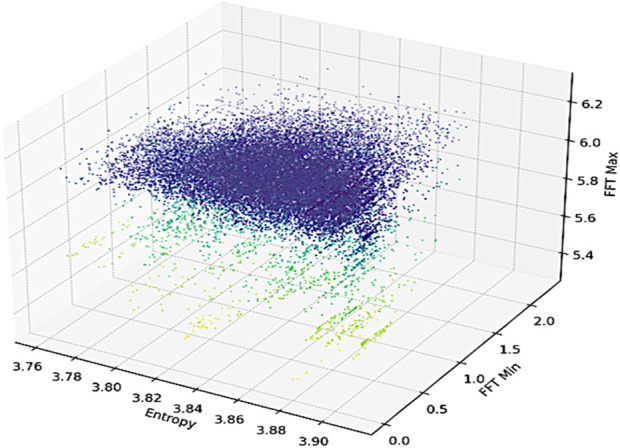
Skewness is calculated from the Opportunity (left) and Extrasensory (right) datasets.

Skewness and kurtosis (Wang et al., 2021; Ramanujam et al., 2019; AlZubi et al., 2014) are statistical measures that describe the shape and characteristics of a distribution. Skewness quantifies the extent and direction of the skew in the data. A negative skew indicates that the left tail is longer, while a positive skew indicates a longer right tail. The mathematical equation for skewness (Yu et al., 2021; Zhang et al., 2021; Qi et al., 2022; Zheng et al., 2023c) is

$$\text{Skewness}=\frac{\sum_{i=1}^{n}{\left(x_{i}-\bar{x}\right)}^{3}}{\left(N-1\right).s^{3}}$$

where $x_{i}$ are the individual sample points, $\bar{x}$ is the sample mean, *s* is the standard deviation (Liu et al., 2021; J, X. et al., 2022; Mao et al., 2022b; Guo et al., 2022; Xu et al., 2022b), and *N* is the number of samples. Figure 7 shows skewness for different activities over both datasets.

#### 3.4.6 kurtosis

Kurtosis (Lu et al., 2023; Liu et al., 2023b; Liu et al., 2023c; Liu et al., 2023d) on the other hand, measures the tailedness of the distribution. Higher kurtosis indicates a more extreme result, meaning that more of the variance is the result of infrequent extreme deviations, as opposed to frequent modestly sized deviations (Miao et al., 2023; Di et al., 2023; Ahmad et al., 2020; Liu et al., 2023e). The mathematical equation for kurtosis is

$$\text{Kurtosis}=\frac{\sum_{i=1}^{N}{\left(x_{i}-\bar{x}\right)}^{4}}{\left(N-1\right).s^{4}}-3$$

**FIGURE 8:**
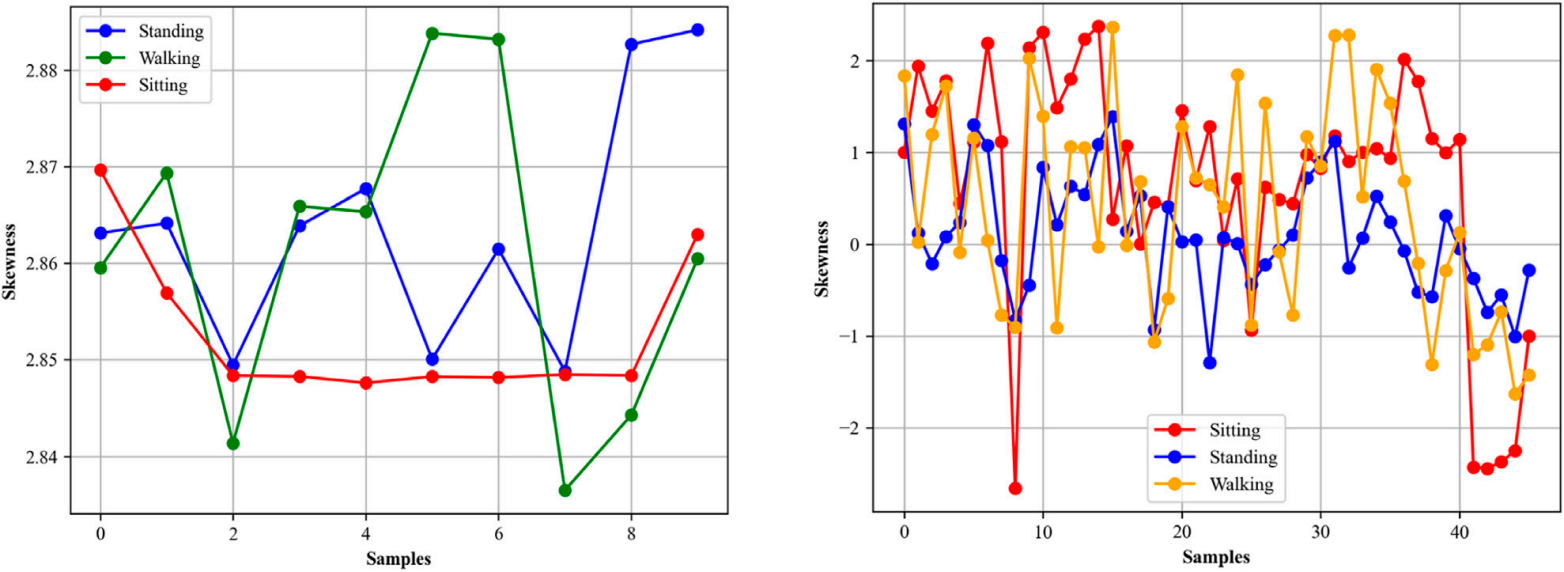
Kurtosis calculated from the Opportunity (left) and Extrasensory (right) datasets.

Both skewness and kurtosis provide valuable information on the nature of variability in a set of numbers and are especially useful in the field of Human Activity Recognition (HAR) to distinguish between different types of activity. Skewness could provide clues about the symmetry of the user’s motion, and kurtosis could indicate the extremity of the user’s activities. We extracted kurtosis for different types of activities in Figure 8.

### 3.5 Feature extraction for location-based activity

For localization activity, we extracted separate features. These features include step count detection, step length calculation (Gu et al., 2017; Kang et al., 2018), and MFFCs. We describe each feature in detail one by one.

#### 3.5.1 Step count detection

In indoor localization and activity recognition, the step count (Sevinç et al., 2020; Gu et al., 2019) emerges as an important metric with multifaceted applications. It serves as a fundamental parameter for activity profiling, aiding in the differentiation of various human movements such as walking, running, or standing. Key features like step count and heading angle are integral to the development of robust and precise indoor localization systems, especially in environments where GPS signals are weak or entirely absent (Zhang and Jiang, 2021; Xu et al., 2023).

The step count was determined using accelerometer data (Pham et al., 2021) collected from the waist of the subject during walking activity. First, we combined the raw acceleration data along the x , y , and z axes to form a composite magnitude signal. This signal was then shifted to ensure that all values were positive. The mean of the shifted signal was calculated, and peaks that exceeded this mean were identified and counted as steps in Supplementary Figure S2. The magnitude of the acceleration *A* was calculated as

$$A=\sqrt{x^{2}+y^{2}+z^{2}}$$

#### 3.5.2 Step length estimation

Step length, or stride length (Ahn and Yu, 2007; Hu et al., 2020) is important in the domain of indoor localization (Yoon and Kim, 2022) and human activity tracking. This metric essentially quantifies the distance covered in a single stride and serves as an essential parameter for accurately estimating an individual’s location within a confined space. We utilized valley points in the position-time curve to estimate the stride length. Valley points in the position-time curve typically represent instances where the same foot hits the ground in successive strides. The curve itself is derived from double-integrating the acceleration data. This method is particularly beneficial in indoor settings, where GPS data may be unreliable or unavailable. Mathematically, the first step involves calculating the velocity *V* by integrating the acceleration A *:*

V=∫Adt

Following this, the position P is calculated by integrating the velocity:

P=∫Vdt

We then identified valley points in this position-time curve. These points are local minima in the curve and represent the moments where a complete stride has occurred, that is, the same foot has hit the ground twice. The time difference between successive valley points is calculated as

$$t_{{valley}_{n}-}t_{{valley}_{n-1}}$$

**FIGURE 9:**
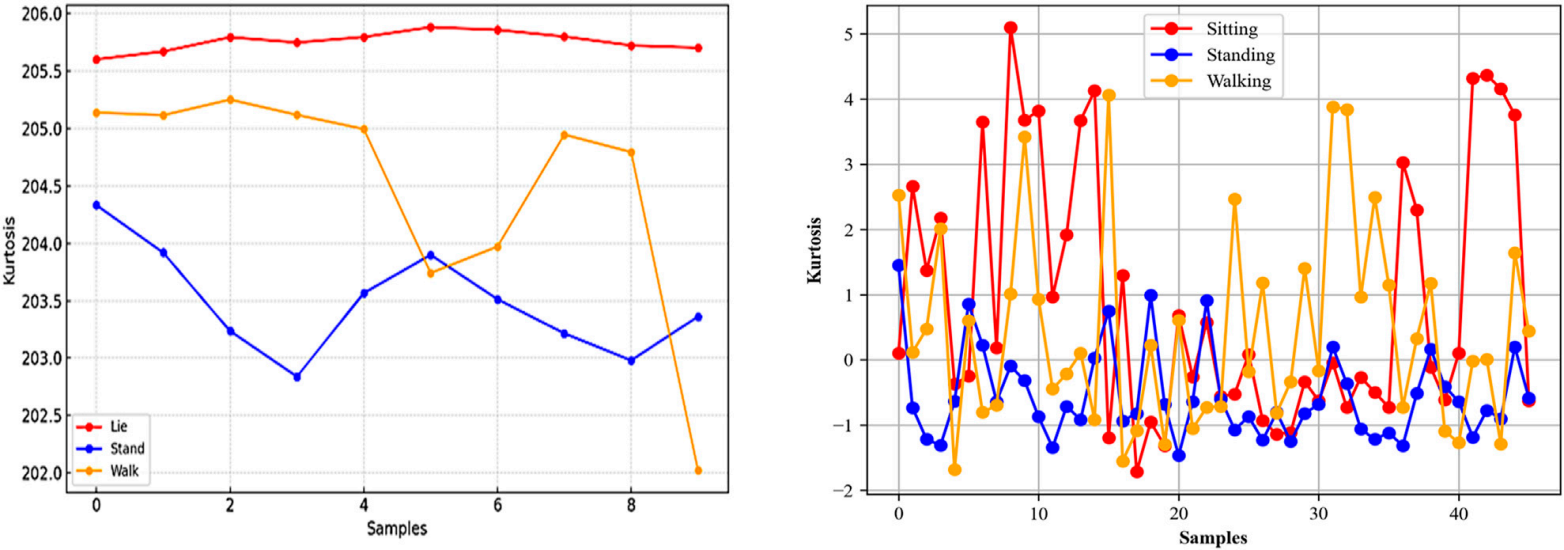
Step length calculated from indoor (left) and outdoor (right) activities over the Extrasensory dataset.

This time difference *Δt*, when multiplied by a constant or average speed, gives the stride length for that particular step. In Figure 9, step lengths calculated for indoor and outdoor activities can be seen intuitively.

#### 3.5.3 Heading angles

The calculation of the heading angle (Javeed and Jalal, 2023; Azmat et al., 2022) is an important component in indoor localization (Jiang et al., 2023), as it provides the orientation or directional information of an individual in relation to Earth’s magnetic North. This orientation data is particularly for accurate path tracking and route reconstruction within indoor environments, where GPS signals are often weak. In our study, the heading angle, was calculated using magnetometer data, which measures the Earth’s magnetic field components along the *x, y,* and *z*-axes. Given that the magnetometer can capture the Earth’s magnetic field, it serves as a reliable sensor for determining orientation. To compute the heading angle, we employed the arctangent function on the *y* and *x* components of the magnetic field as per the following equation:

$$\theta =\arctan2\left(\frac{M_{y}}{M_{x}}\right)$$

The resulting angle *θ* was calculated in radians and later converted to degrees for easier interpretation and application. This angle gives us an understanding of the individual’s orientation at any given point in time, significantly enhancing indoor localization systems. Supplementary Figure S3 displays the heading angles for indoor and outdoor activities over the Extrasensory dataset.

#### 3.5.4 Mel-frequency cepstral coefficients (MFCCs)

The Mel-frequency cepstral coefficients (MFCCs) (González et al., 2015; Hou et al., 2023) are widely used in various applications. They serve as a compact representation of an audio signal, capturing essential characteristics while ignoring less informative variations. In the context of location recognition, MFCCs can help distinguish between different types of environments based on ambient noise or specific sound patterns. For instance, an indoor location might exhibit different MFCC patterns compared to an outdoor location due to the presence of echoes, HVAC noise, or human activity. MFCCs are computed through a series of transformations. We already segmented the audio data in section B. Each segment is passed through an FFT to obtain its frequency spectrum. Then we applied a set of Mel-filters to the frequency spectrum to capture the human perception of pitch. The logarithm of the energies of these Mel-frequencies is then taken, and a discrete cosine transform (DCT) is applied to the log energies. The resulting coefficients are the MFCCs. The equation for the $k^{th}$ MFCC ( $c_{k}$ ) can be summarized as

$$c_{k}=\sum_{n=1}^{N}\log\left(MF_{n}\right)\cos\left(\frac{\pi k\left(n-0.5\right)}{N}\right)$$

where $MF_{n}$ ​ is the Mel-filtered energy of the $n^{th}$ frequency bin, and *N* is the total number of Mel-filters. The MFFCs calculated for indoor and outdoor activities over the Extrasensory dataset can be seen in Supplementary Figure S4.

### 3.6 Feature optimization using Yeo-Johnson power transformation

**FIGURE 10:**
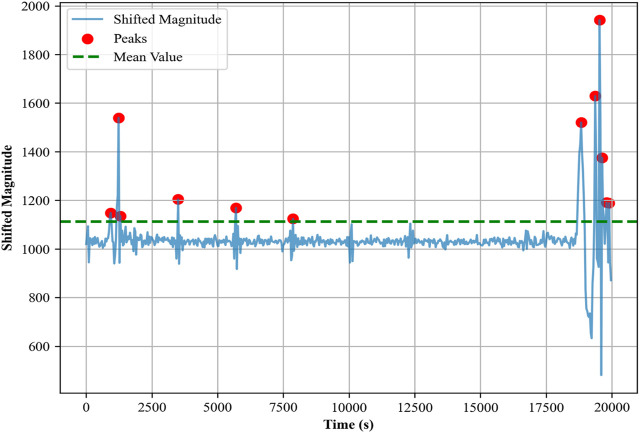
**(A)** original feature vector; **(B)** optimized feature vector over the Extrasensory dataset.

The Yeo-Johnson transformation (Xu et al., 2023) is a power transformation technique aimed at making the data more closely follow a Gaussian distribution, thereby optimizing its characteristics for further analysis. The transformation is similar to the Box-Cox transformation, but it has the advantage of handling negative numbers as well. We started by extracting various features from the time-series data. Each of the features serves as a column in our feature matrix. To apply the Yeo-Johnson transformation, we used the **PowerTransformer** class from the **sklearn. preprocessing** package, which internally fits the optimal λ for each feature based on the likelihood maximization. After fitting, the transformation was applied to each feature vector to create an optimized feature set. Here it is important to note that after optimization, we got two feature vectors, one for localization activities and the second for locomotion activities. We plotted two feature vectors the original *versus* optimized for Walking, Sitting, and Lying activities using only a few features including (Alazeb et al., 2023), FFT-Min/Max, Shannon entropy, and Kurtosis over the Extrasensory dataset in Figure 10. The transformation is defined as

$$\overset{´}{y_{i}=}\left\{\begin{array}{c} \frac{\left[{\left(y_{i}+1\right)}^{\gamma }-1\right]}{\gamma },\text{ if}\;y_{i}\geq 0\;\text{and}\;\gamma \neq 0,\\ \log\left(y_{i}+1\right),\text{if }y_{i}\geq 0\;\text{and}\;\gamma =0,\\ \frac{-\left[{\left(-y_{i}+1\right)}^{\gamma }-1\right]}{\gamma },\text{if }y_{i}<0\;\text{and}\;\gamma \neq 2,\\ -\log\left(y_{i}+1\right),\text{if }y_{i}<0\;\text{and}\;\gamma =2. \end{array}\right.$$

Here, $y_{i}$ is the $i^{th}$ observation, $\overset{´}{y_{i}}$ is the transformed value, and λ is the transformation parameter. It is important to mention that the Yeo-Johnson transformation is often determined by optimizing a likelihood function to find the best λ that minimizes the deviation from normality. The objective function for this is usually the negative log-likelihood, given by

$$L\left(\gamma \right)=-\log\left(\prod_{i=1}^{n}\int \left(\frac{\overset{´}{y_{i}}}{\gamma }\right)\right)$$

where $\int (\frac{\overset{´}{y_{i}}}{\gamma })$ is the probability density function of the transformed data.

### 3.7 Feature evaluation analysis and comparisons

In order to validate and evaluate the robustness of the proposed feature set, we compare the extracted features in this study with other latest existing state-of-the-art methods. Initially, we categorize all features into different sets, from the latest SOTA systems (Bennasar et al., 2022; Tian et al., 2019; Muaaz et al., 2023). The features are partitioned into 4 sets. Each set is subjected to model training and validation. Our observations indicate that our proposed feature set outperforms other sets in performance, thereby validating the effectiveness, robustness, and novelty of our proposed features in enhancing model performance. The details of feature sets and their description are given in Table 1.

**TABLE 1:** Comparison of the proposed feature extraction with the latest SOTA.

Source	Features	Accuracy (%)
Bennasar et al. (2022)	Mean, standard deviation, root mean square, autocorrelation, permutation entropy, etc.	76
Tian et al. (2019)	Mean, variance, skewness, kurtosis, signal magnitude area, minimum/maximum, interquartile range, etc.	83
Muaaz et al. (2023)	Mean, variance, skewness, kurtosis, entropy, total energy, slope, etc.	88
Proposed	Skewness, kurtosis, phase angle, linear prediction, auto-regression etc.	96

## 4 Experimental setup and datasets

### 4.1 Experimental setup

The research experiments were carried out on a laptop equipped with an Intel Core i5-8500U processor running at 3.10 GHz, 16.0 GB of RAM, and the Mac operating system. The Jupyter Notebook was utilized as the primary programming environment. We conducted comprehensive experiments using two widely recognized benchmark datasets, Opportunity and Extrasensory. The Opportunity dataset, a renowned benchmark in the field, captures data from various sensors. Another dataset used in our research is the Extrasensory dataset. With its rich sensory data, it offers an extensive range of human locomotion and localization activity. The time-series data was partitioned into approximately equal-length segments for the purpose of cross-validation (Xu et al., 2022a). In each of the k iterations, k−1 segments were designated for training, and the remaining segment was set aside for testing. This procedure was repeated k times, guaranteeing that each segment served as a test set once, the rest being used as training sets. Importantly, we maintained a strict separation between training and test sets in every iteration, preventing any overlap or data sharing between them.

### 4.2 Dataset description

In the subsequent subsection, we provide comprehensive and detailed descriptions of each dataset used in our study. Each dataset is thoroughly introduced, highlighting its unique characteristics, data sources, and collection methods.

#### 4.2.1 The opportunity dataset

The Opportunity dataset (Lukowicz et al., 2010) stands as a key benchmark in the domain of human activity recognition. It was collected from 12 subjects participating in various daily activities, ensuring a diverse representation. The dataset captures data from different sensors, such as accelerometers, gyroscopes, and magnetometers, strategically positioned on the participants’ bodies and on certain daily-use objects. These sensors record data during both dynamic and static human activities. The dynamic activities include actions like walking, jogging, and opening doors, while the static activities encompass states like standing, sitting, and lying down. Additionally, there are more complex activities, like making coffee or preparing a sandwich, which involve interactions with objects and the environment. In total, the Opportunity dataset covers 17 different activities, ranging from basic locomotion tasks to more intricate, multi-step actions. These activities were recorded in diverse scenarios, both scripted and unscripted, to ensure a comprehensive representation of real-world conditions.

#### 4.2.2 The extrasensory dataset

The Extrasensory dataset (Vaizman et al., 2017) is a robust collection of data sourced from 60 distinct participants, each uniquely identified by a UUID. These participants contributed thousands of data samples. While the majority of these samples were recorded at consistent 1-min intervals, there are instances where time gaps exist. Each data sample encompasses measurements derived from a variety of sensors present in the participants’ personal smartphones and a provided smartwatch. Furthermore, a large portion of these data points come furnished with context labels, as self-reported by the individual participants. In terms of device usage, the dataset includes data from 34 iPhone users and 26 Android users. The gender distribution is fairly balanced, with 34 females and 26 males. Sensors integrated into the dataset include accelerometer, gyroscope, magnetometer (He and Jin, 2008), GPS, audio, compass, and smartwatch sensors. The dataset provides a variety of human activities, including indoor, outdoor, transportation, and locomotion.

## 5 Results and analysis

In this section, we performed different experiments for the proposed system. The system is evaluated using different matrices, including confusion matrix, precision, recall, F1 score and receiver operating characteristic (ROC) curve. The detailed discussion and analysis are described below.

### 5.1 Confusion matrices for locomotion activities

**FIGURE 11:**
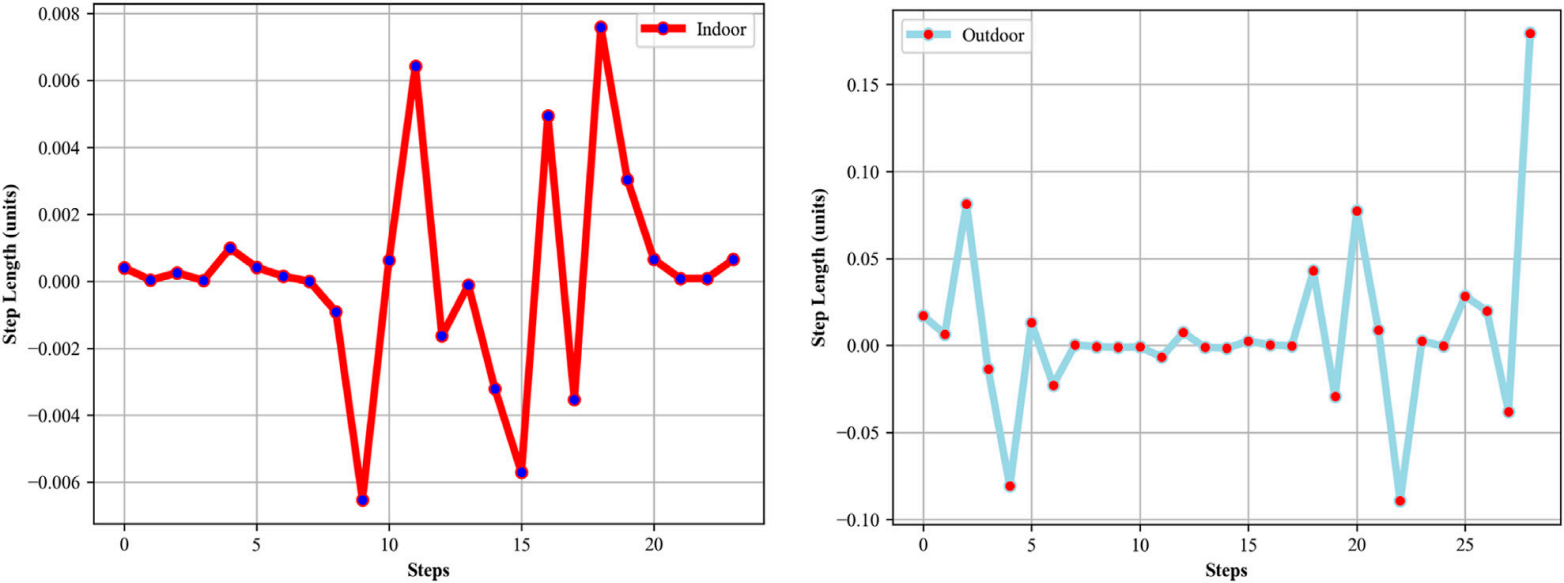
Confusion matrix: locomotion activities in the Extrasensory dataset.

**FIGURE 12:**
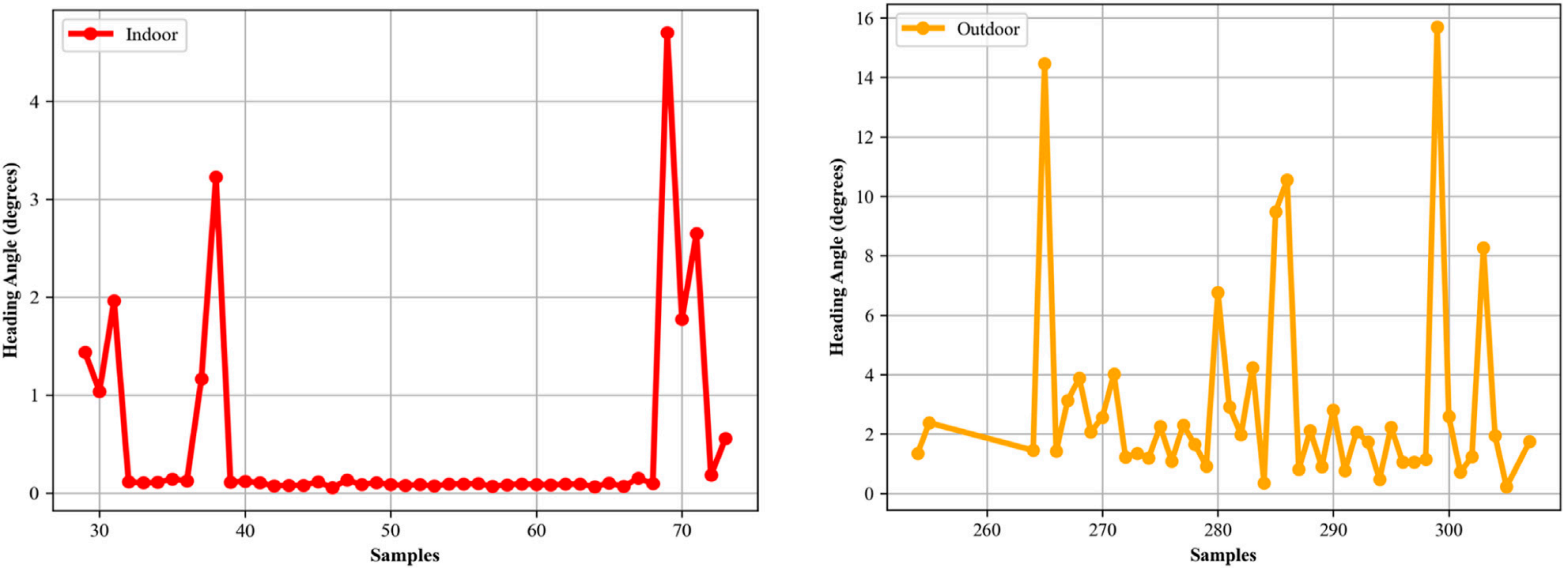
Confusion matrix: locomotion activities in the Opportunity dataset.

We assessed our system’s performance for locomotion activities across both datasets. Impressively, the system achieved a 97% accuracy rate on the Opportunity dataset and 89% on the Extrasensory dataset. Figures 11, 12 present the confusion matrices for both datasets.

The system shows high performance in identifying stationary activities. The system shows excellent performance in correctly identifying Sitting and Standing, with accuracies of 97% and 99%, respectively. This suggests that the system is highly effective in recognizing stationary activities. This capability is particularly applicable in contexts like workplace ergonomics or patient monitoring, where it is important to track the amount of time spent sitting or standing.

#### 5.1.1 Moderate performance in dynamic activities

The performance in recognizing Walking is moderate, with an accuracy of 67%. The system seems to confuse Walking with Lying Down in some cases, which might be due to similar sensor patterns during slow walking or transitional movements. This indicates a potential area for improvement, especially in applications like fitness tracking or elderly care, where accurate recognition of walking is crucial.

#### 5.1.2 Strong recognition of lying down

The system accurately identifies Lying Down in 96% of the cases, indicating its effectiveness in distinguishing this activity from others. This could be particularly relevant in healthcare applications, like patient monitoring systems, where detecting prolonged periods of lying down is important.

#### 5.1.3 Near-perfect recognition of all activities in the opportunity dataset

The system shows near-perfect accuracy in recognizing all four activities: Standing, Walking, lying, and sitting, with accuracies of 100%, 98%, 96%, and 95% respectively. This high level of accuracy is significant for applications that require precise activity recognition, such as in advanced assistive technologies or smart home environments.

#### 5.1.4 Applicability across diverse scenarios

Given the high accuracy in all activities, this system can be confidently applied to diverse real-world scenarios, from fitness tracking to elderly care, where accurate activity recognition is crucial. The system’s ability to distinguish between similar activities (like lying and sitting) demonstrates its sophistication and reliability.

#### 5.1.5 General observations

The higher overall mean accuracy in the Opportunity dataset (97.25%) compared to the Extrasensory dataset (89.75%) could be attributed to differences in sensor quality, data collection protocols, or the inherent nature of the activities in each dataset. The system’s performance on the Opportunity dataset suggests its potential effectiveness in environments with structured activities, while the Extrasensory dataset results indicate the need for refinement in more complex or less structured environments.

### 5.2 Precision, recall, and F1 score values for locomotion activities

We continued to investigate in more depth the evaluation of our system using precision, recall, and F1 score. Across both datasets, the system demonstrated strong performance in all of these metrics. Tables 2 and 3 showcase the system’s performance.

**TABLE 2:** Precision, recall, and F1 score: locomotion activities in the Extrasensory dataset.

Classes	Precision	Recall	F1 score
Sitting	0.97	0.98	0.97
Walking	1.00	0.33	0.50
Lying Down	0.96	0.93	0.95
Standing	0.88	1.00	1.00
Macro-average	0.95	0.81	0.85

**TABLE 3:** Precision, recall and F1 score: locomotion activities in the Opportunity dataset.

Classes	Precision	Recall	F1 score
Standing	1.00	1.00	1.00
Walking	1.00	0.95	0.99
Lie	0.99	0.98	0.99
Sitting	1.00	1.00	1.00
Macro-average	0.99	0.98	0.99

The performance evaluation of our system on the Extrasensory and Opportunity datasets, as reflected in Tables 4 and 5, highlights its strengths and areas for improvement in activity recognition. In the Extrasensory dataset, the system exhibits high precision across all activities, particularly for ‘Sitting’ and ‘Lying Down’, with scores of 0.97 and 0.96, respectively. This indicates a strong capability to correctly identify these activities when they occur. However, there is a notable discrepancy in the recall for ‘Walking’, at only 0.33, despite a perfect precision score. This suggests that while the system is accurate when it detects walking, it frequently misses walking instances. The overall macro-average scores of 0.95 for precision and 0.81 for recall, with an F1 score of 0.85, reflect competent performance but highlight the need for improvements in consistently recognizing walking activities. In contrast, the system’s performance on the Opportunity dataset is exemplary, achieving near-perfect scores across all activities. Precision and recall are both 1.00 for ‘Standing’, ‘Walking’, and ‘Sitting’, with ‘Lie’ closely following at 0.99 for both metrics. This exceptional performance, encapsulated in macro-average scores of 0.99 for both precision and recall, and an F1 score of 0.99, demonstrates the system’s high efficacy in structured environments with clear activity definitions.

**TABLE 4:** Precision, recall, and F1 Score: localization activities in the Extrasensory dataset.

Classes	Precision	Recall	F1 score
Indoors	1.00	1.00	1.00
At School	0.88	1.00	0.93
Location Home	1.00	1.00	1.00
Location Workplace	1.00	1.00	1.00
Outside	1.00	0.93	0.96
Macro-average	0.97	0.98	0.97

**TABLE 5:** Comparisons of the proposed system with other systems.

Methods	Opportunity	Extrasensory
Javeed, M. et al. (2023)	0.88	-
Vanijkachorn and Visytsak (2021)	0.88	-
Han, J. (2019)	0.87	-
Gil-Martin, et al. (2020b)	0.67	-
Gioanni et al. (2016)	0.74	-
Vaizman et al. (2018)	-	0.83
Asim et al. (2020)	-	0.87
Abduallah et al. (2022)	-	0.87
Proposed system mean accuracy	0.97	0.96

### 5.3 Receiver operating characteristic curves for locomotion activities

To further investigate the system and stability of the system, we evaluated the system using the roc curve. The receiver operating characteristic (ROC) curve is a graphical representation used to evaluate the performance of a classifier across various decision thresholds. An important aspect of the ROC curve is the area under the curve (AUC). The AUC provides a single-number summary of the classifier’s performance. A value of 1 indicates perfect classification, while a value of 0.5 suggests that the classifier’s performance is no better than random guessing. In Supplementary Figures S5, S6, the ROC curves for both datasets can be observed clearly.

#### 5.3.1 The Opportunity dataset

Standing (AUC = 1.00): The model’s perfect score in identifying standing activities underscores its precision in environments such as elderly care, where detecting prolonged stationary periods is crucial for monitoring wellbeing and preventing health risks.

Walking (AUC = 0.98): The high AUC value for walking reflects the model’s strong capability in accurately tracking walking movements, which is particularly beneficial for applications in fitness tracking and urban navigation systems.

Lying (AUC = 0.99): This near-perfect score indicates the model’s effectiveness in recognizing lying down postures, an essential feature for patient monitoring in healthcare settings, especially for bedridden individuals.

Sitting (AUC = 1.00): The model’s flawless detection of sitting activities is critical to workplace ergonomics and sedentary lifestyle analysis, aiding in developing interventions for prolonged inactivity.

#### 5.3.2 The extrasensory dataset

Sitting (AUC = 0.97): The high AUC for sitting activities demonstrates the system’s reliability in identifying sedentary behaviors, which is vital in office settings for promoting active work habits.

Lying Down (AUC = 0.96): This score reflects the model’s adeptness in detecting lying down positions, applicable in sleep analysis and residential healthcare monitoring.

Walking (AUC = 0.67): The lower AUC in this category suggests challenges in distinguishing walking from other movements in complex environments, pointing to potential areas for improvement in applications requiring precise motion tracking.

Standing (AUC = 0.99): The high accuracy in identifying standing positions is crucial in varied contexts such as retail analytics and customer behavior studies, where understanding patterns of movement and pause can enhance service strategies.

### 5.4 Confusion matrix for localization activities

We conducted experiments to recognize localization activities. These experiments were conducted using the extrasensory dataset, which offers a variety of human localization activities. Initially, we generated a confusion matrix, followed by an assessment of the system’s performance using precision, recall, and the F1 score. Moreover, to assess the system’s stability and effectiveness, we employed the ROC curve. Each experiment is thoroughly discussed, and the resulting outcomes are presented below.

**FIGURE 13:**
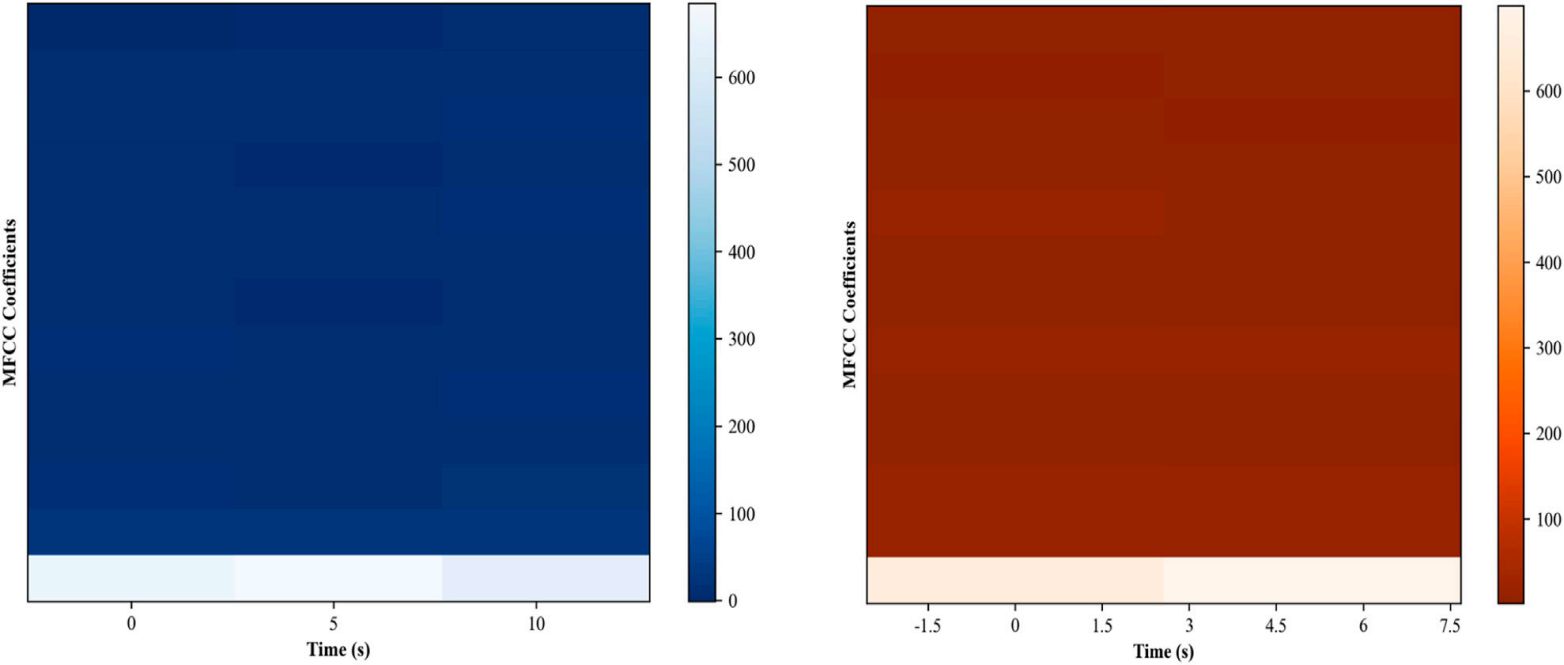
MFCCs feature.

In this experiment, we evaluate the system for localization activities. We observed a good accuracy rate of 96%. The confusion matrix is given in Figure 13.

The confusion matrix for the Extrasensory dataset’s localization activities provides valuable insights into the system’s capability to accurately distinguish between different environmental contexts. Indoors (accuracy = 100%): The perfect accuracy in identifying indoor activities showcases the model’s precision in environments like homes, offices, or malls. This is crucial for applications such as smart home automation, where accurate indoor localization can trigger context-specific actions like adjusting lighting or temperature. At School (accuracy = 90%): The high accuracy in recognizing activities at school is significant for educational applications, such as attendance tracking or student activity monitoring. The confusion with other locations, although minimal, suggests room for improvement in differentiating between similar educational and other public environments. Location Home (accuracy = 100%): Flawless detection of activities at home points to the model’s effectiveness in residential settings, crucial for applications like security systems or elder care monitoring, where distinguishing home activities is essential for providing personalized and situational services. Outside (accuracy = 100%): The model’s ability to perfectly identify outdoor activities is vital for location-based services, such as navigation aids and outdoor fitness tracking. This can enhance user experiences in applications that rely on outdoor localization. Location Workplace (accuracy = 94%): The high accuracy in identifying workplace activities is important for enterprise solutions, like workforce management and safety compliance monitoring. The slight confusion with other locations highlights the need for further refinement to distinguish workplace activities from similar environments with greater accuracy.

### 5.5 Precision, recall, and F1 score values for localization activities

To check the performance of the system for localization activities, we calculated the precision, recall, and F1 score. In Table 4.

### 5.6 Receiver operating characteristic curve for localization activities

We plotted the ROC curve of localization activities to ensure that the proposed system is well trained, accurate, and stable. The system showed very impressive results in recognizing location-based activities. The ROC curve can be examined in Supplementary Figure S7.

### 5.7 Detailed performance analysis

In this subsection, we delve deeper into the performance metrics across different datasets and activities, shedding light on the trade-offs between accuracy and computational efficiency.

#### 5.7.1 Locomotion activities

For the Opportunity dataset, as the number of iterations increased from 5 to 50, the accuracy improved from 74.76% to 97.14%, while the computation time increased from 2.53 s to 19.49 s. For the Extrasensory dataset’s locomotion activity, accuracy improved from 61.76% at 5 iterations to 89.14% at 50 iterations, with computation time increasing from 1.49 s to 14.49 s. It is evident that the model trained on the Opportunity dataset achieved a relatively higher accuracy with more iterations compared to the Extrasensory dataset. However, the computational cost was also higher for the Opportunity dataset. The time complexity and efficiency plot can be seen in Supplementary Figure S8.

#### 5.7.2 Localization activities

For the Extrasensory dataset’s localization activity, the accuracy improved from 85.76% at 10 iterations to 95.61% at 50 iterations. The corresponding computation time rose from 1.93 s to 11.90 s. The model’s accuracy for localization showed a steady improvement with increased iterations, and the computational cost was relatively consistent, indicating efficient model performance. The time complexity and efficiency plot for the localization activities can be seen in Supplementary Figure S9.

### 5.8 Comparison between locomotion and localization activities

For the Extrasensory dataset, the model’s performance for localization activities was consistently higher in terms of accuracy compared to locomotion activities, across the same number of iterations. However, the computation time for localization was slightly longer, indicating a trade-off between accuracy and computational efficiency.

Finally, our system was subject to a comparative analysis against other existing systems, revealing that our model excels in terms of accuracy. Table 5 provides a comprehensive overview of the comparisons between our system and state-of-the-art techniques.

## 6 Discussion

A system for recognizing human locomotion and location-related activities is introduced in this work. This system utilizes advanced noise filtering techniques, signal segmentation methods, feature extraction processes, and hybrid models to effectively identify both locomotion and localization activities. It is designed to be versatile and can find applications in various real-world scenarios such as sports, healthcare, security, location recognition, and many more real-world applications. Our system, while advanced, has certain limitations. The sensors we utilized, especially GPS and IMU, have inherent challenges; GPS may not always be accurate indoors or amidst tall urban structures, and IMUs can drift over time. Second, our reliance on the Opportunity and Extrasensory datasets, although reputable, does not capture all human activity nuances, as evidenced by the challenge of recognizing walking activities. Additionally, our experiments were conducted on a specific laptop configuration. When transitioning to real-world wearable devices, differing computational capabilities might influence the system’s performance. Moving forward, we plan to enhance our system by exploring advanced sensor fusion techniques, allowing for more robust data integration from various sensors. We also recognize the need to diversify our datasets and will seek collaborations to gather more varied and balanced human activity data. Importantly, to ensure our system’s efficacy on wearable devices, we will explore optimizations customized to devices with varied computational constraints, ensuring our HAR system remains efficient and real-time in practical scenarios.

## 7 Conclusion

This study introduces a new and resilient system designed to identify human locomotion and localization activities effectively. The system was developed with a focus on utilizing advanced techniques such as sensor data filtering, windowing, and segmentation, along with innovative methods for feature extraction. It is important to mention that our primary emphasis was on recognizing localization activities, for which we employed robust feature extraction techniques, including step count, step length, and heading angle. In addition to manual feature extraction, we introduced a hybrid model that harnesses both machine learning and deep learning approaches to enhance accuracy in recognition tasks. As a result, the presented system demonstrates precise and efficient recognition of both locomotion and localization activities.

## Data Availability

Publicly available datasets were analyzed in this study. This data can be found here: The Opportunity Dataset: https://archive.ics.uci.edu/dataset/226/opportunity+activity+recognition; The ExtraSensory Dataset: http://extrasensory.ucsd.edu/.
