# Supplementary material for: Robust human locomotion and localization activity recognition over multisensory
Source: Front Physiol. 2024 Feb 21;15:1344887. doi: 10.3389/fphys.2024.1344887 (PMC10915014; doi:10.3389/fphys.2024.1344887)
Supplement: Supplementary file 1 [file DataSheet1.docx]

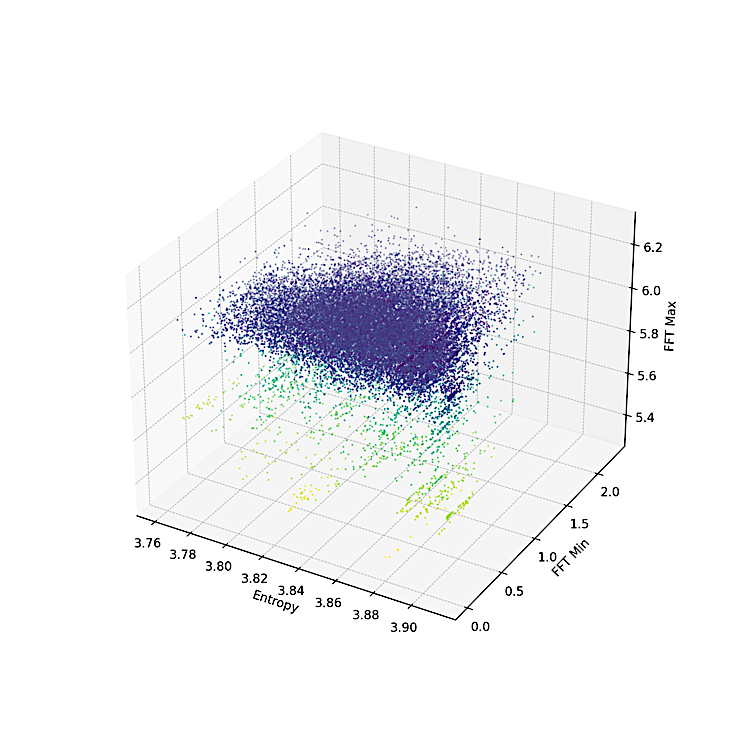


Figure S1. FFT-MIN/MAX and Shannon entropy were calculated from the Opportunity and Extrasensory datasets.

**
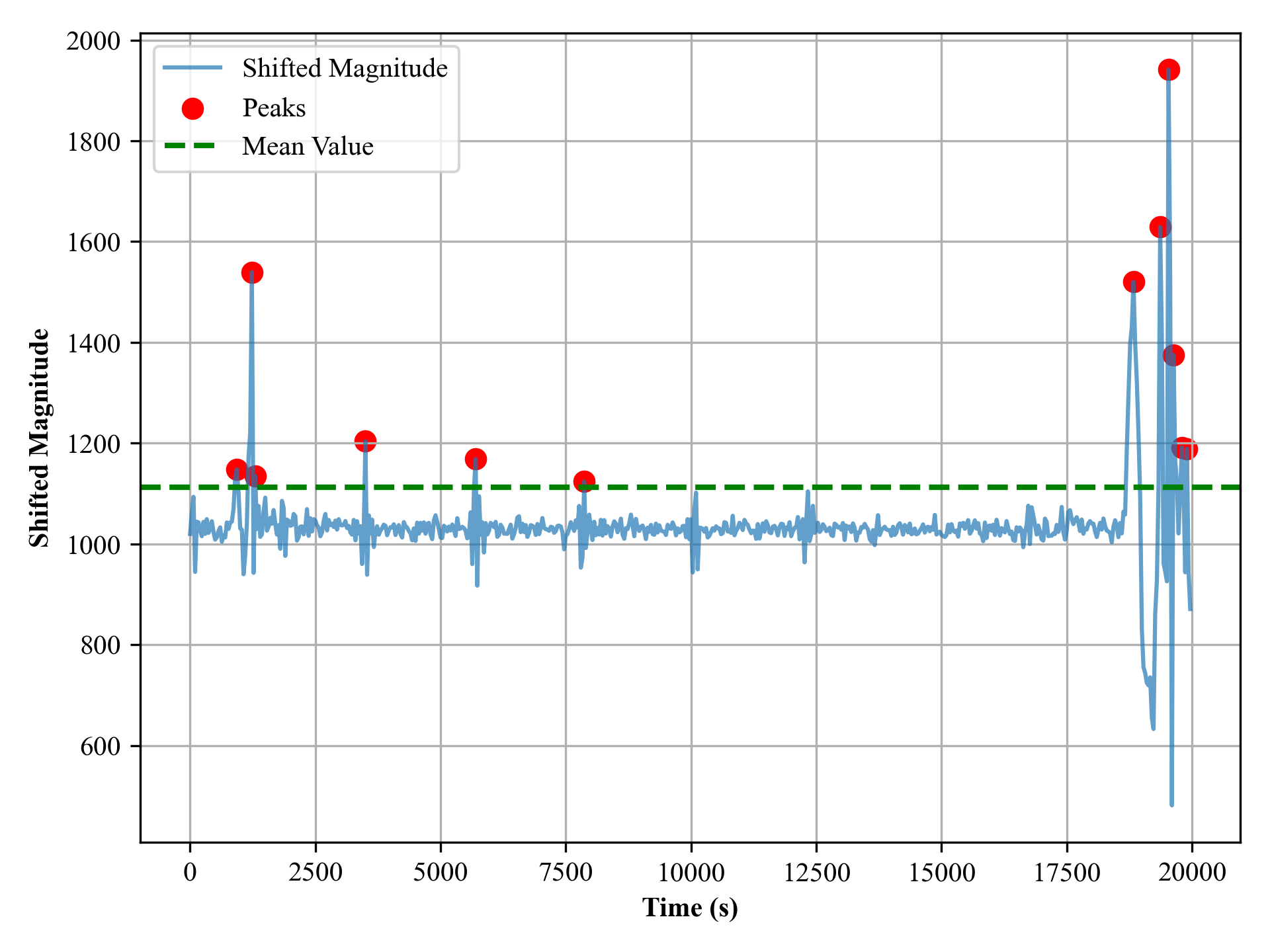
**

Figure S2. Step detected from the activity “walking” over the Extrasensory dataset.


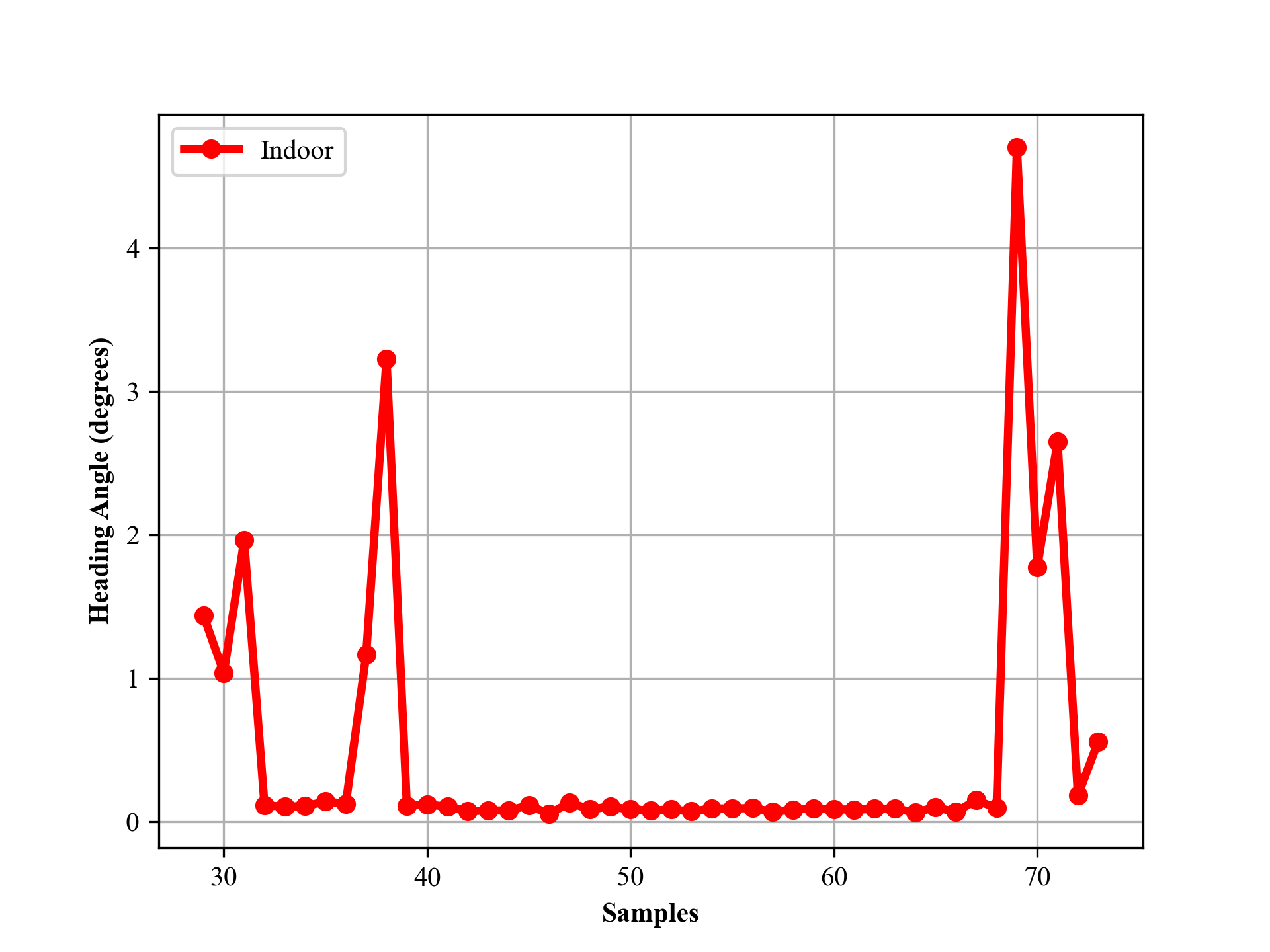

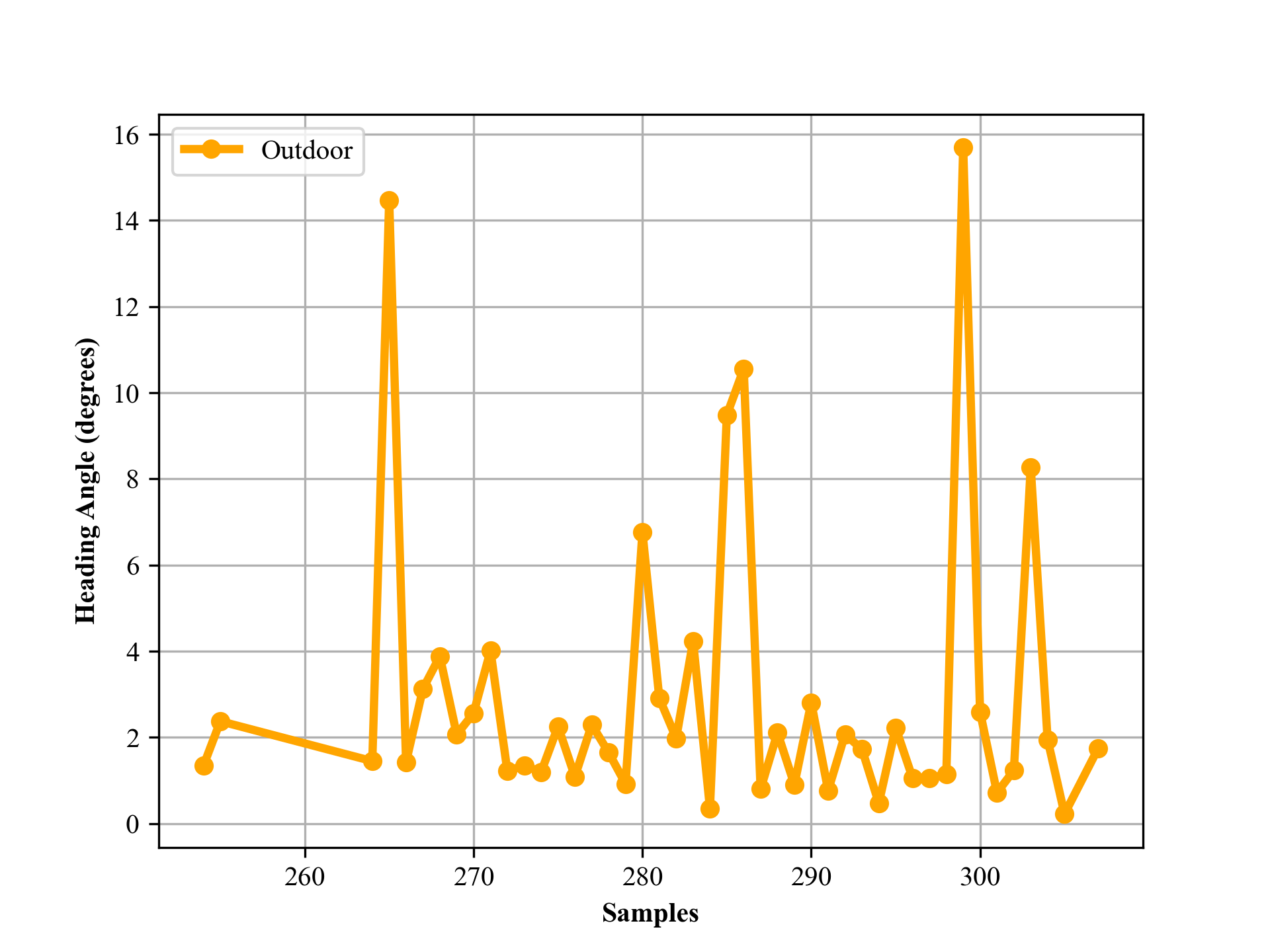


Figure S3. Heading angles for indoor (left) and outdoor (right) activities over the Extrasensory dataset.


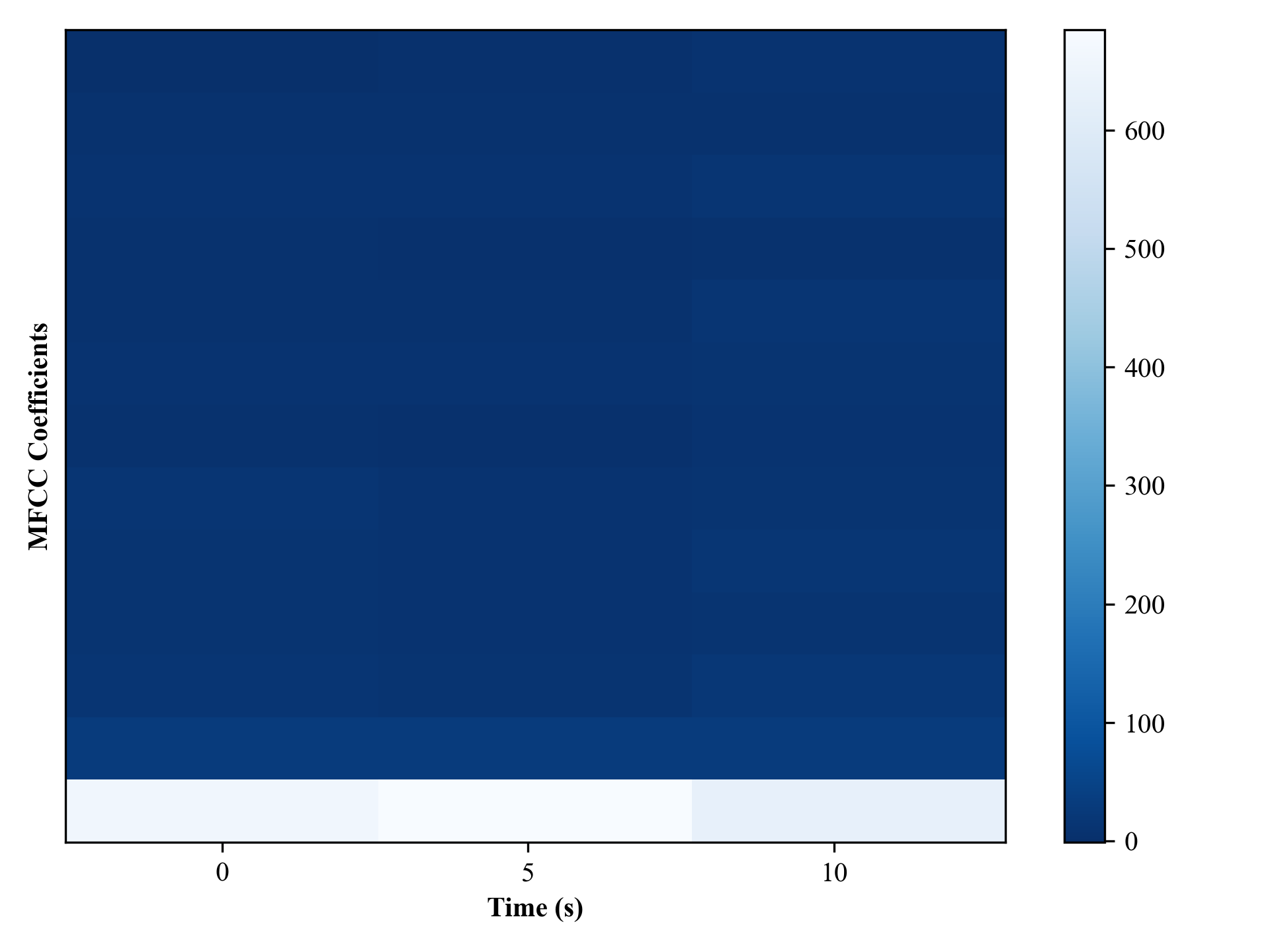

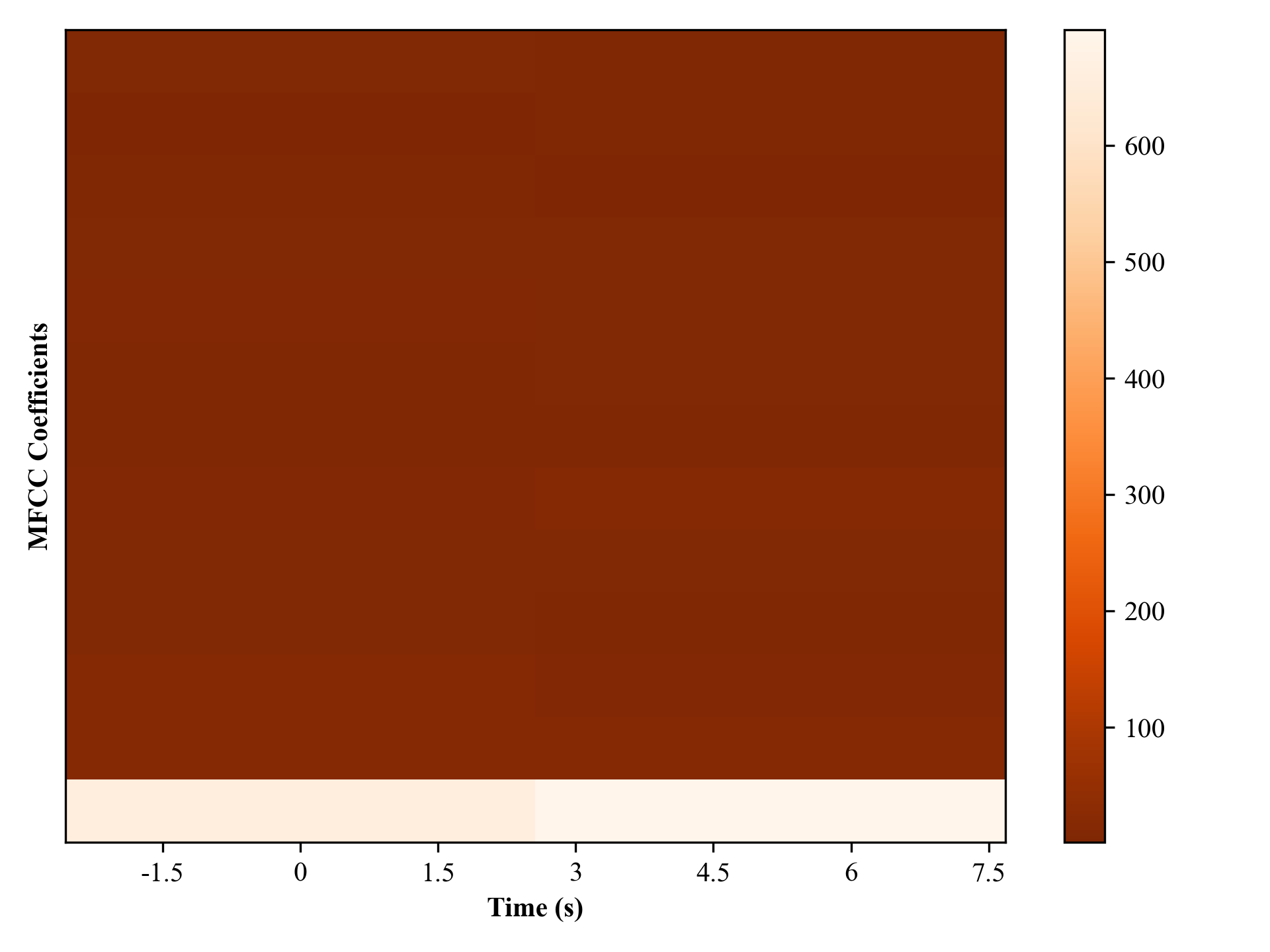


Figure S4. MFFCs for outdoor (left) and indoor (right) activities over the Extrasensory dataset.


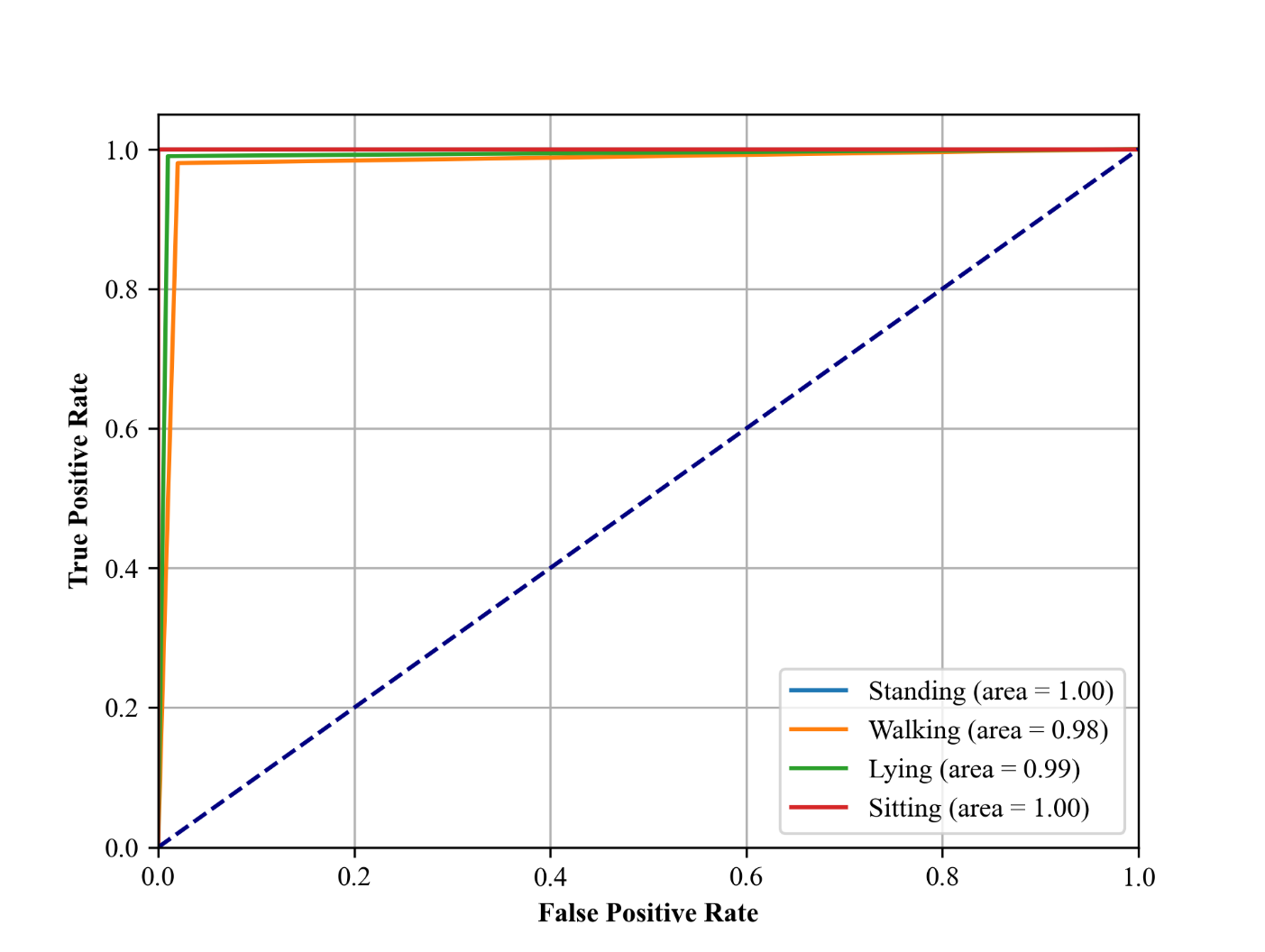


Figure S5. ROC curve: locomotion activities in the Opportunity dataset.


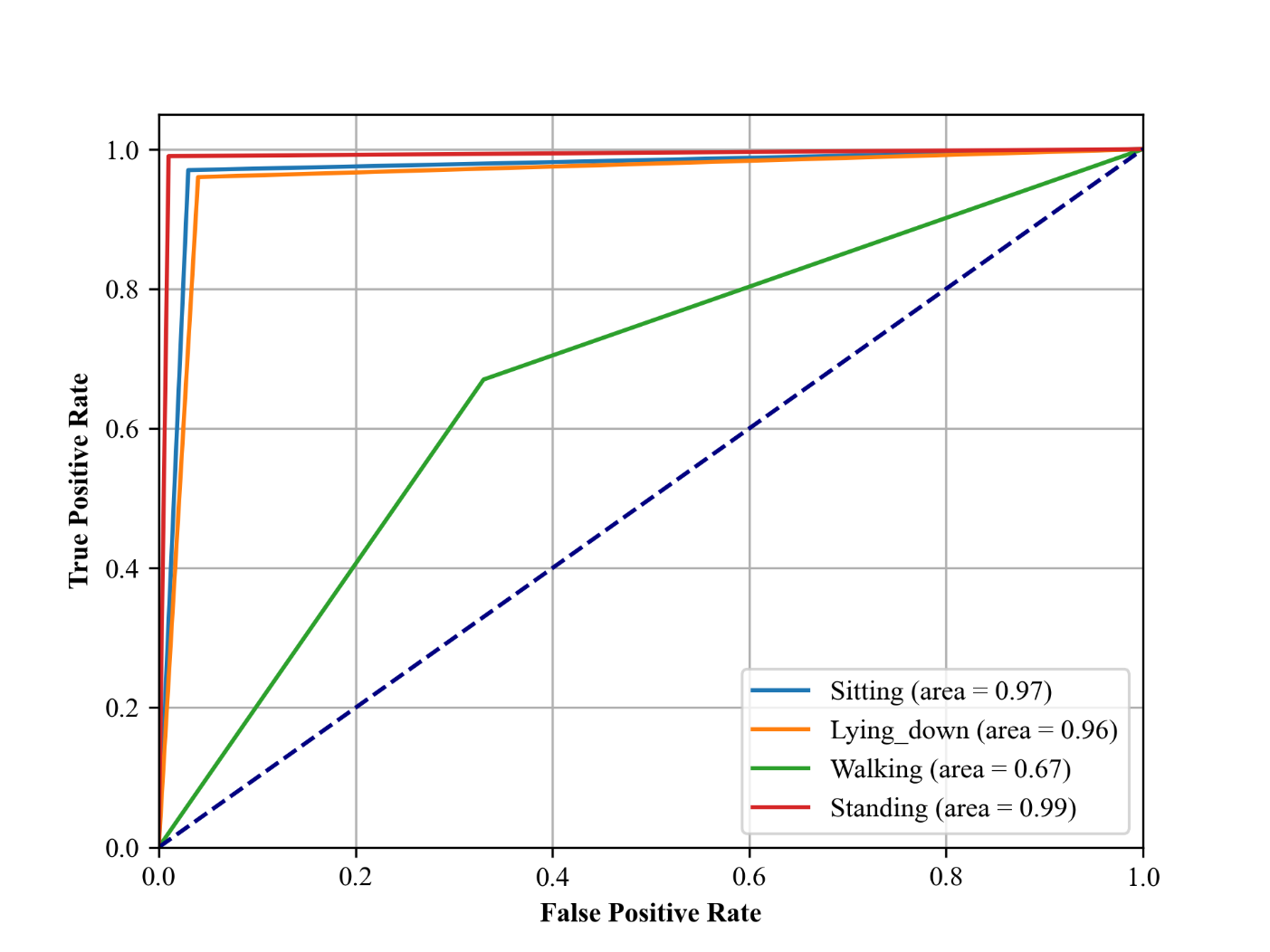


Figure S6. ROC curve: locomotion activities in the Extrasensory dataset.


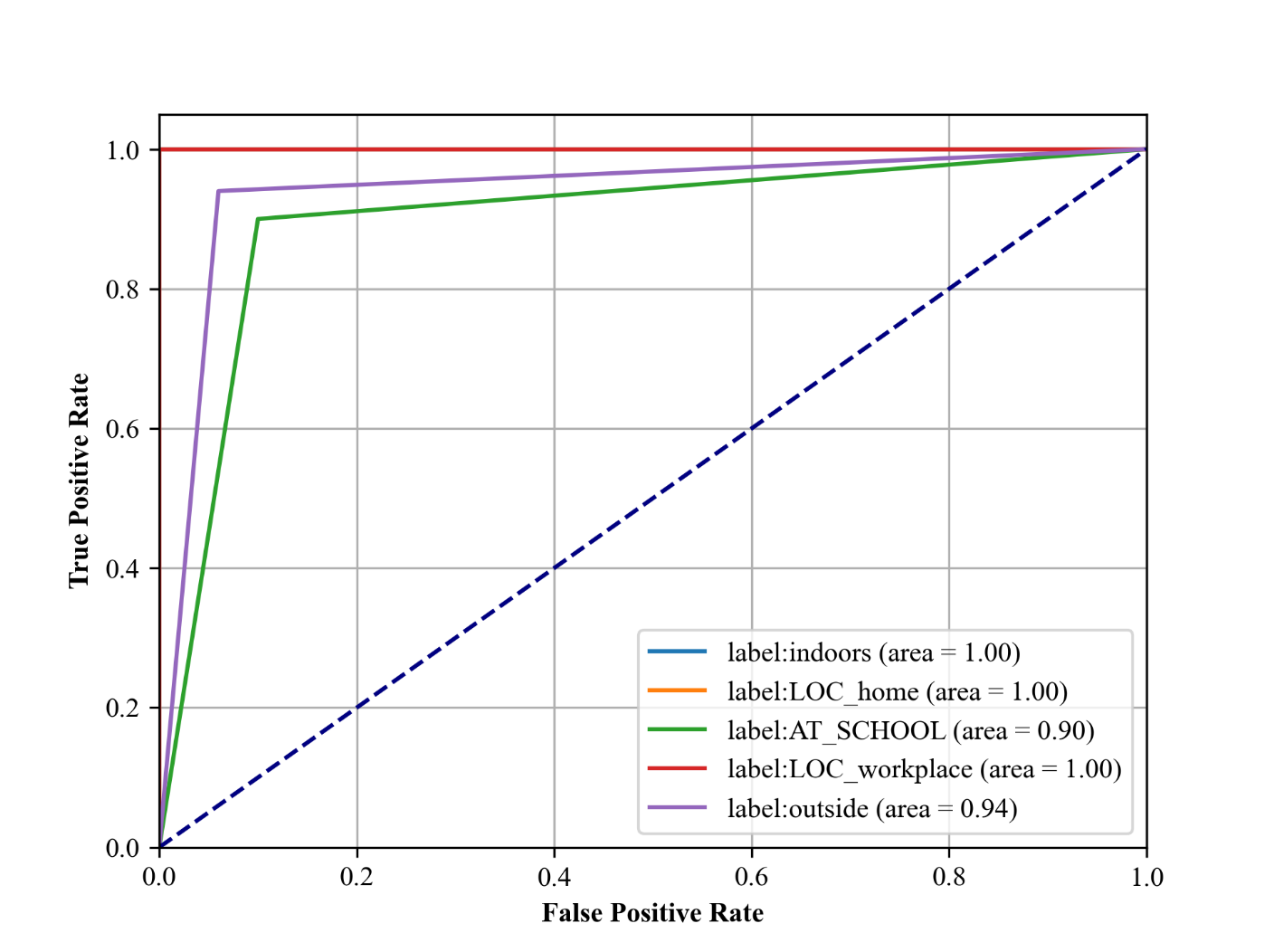


Figure S7. ROC curve: localization activities in the Extrasensory dataset.


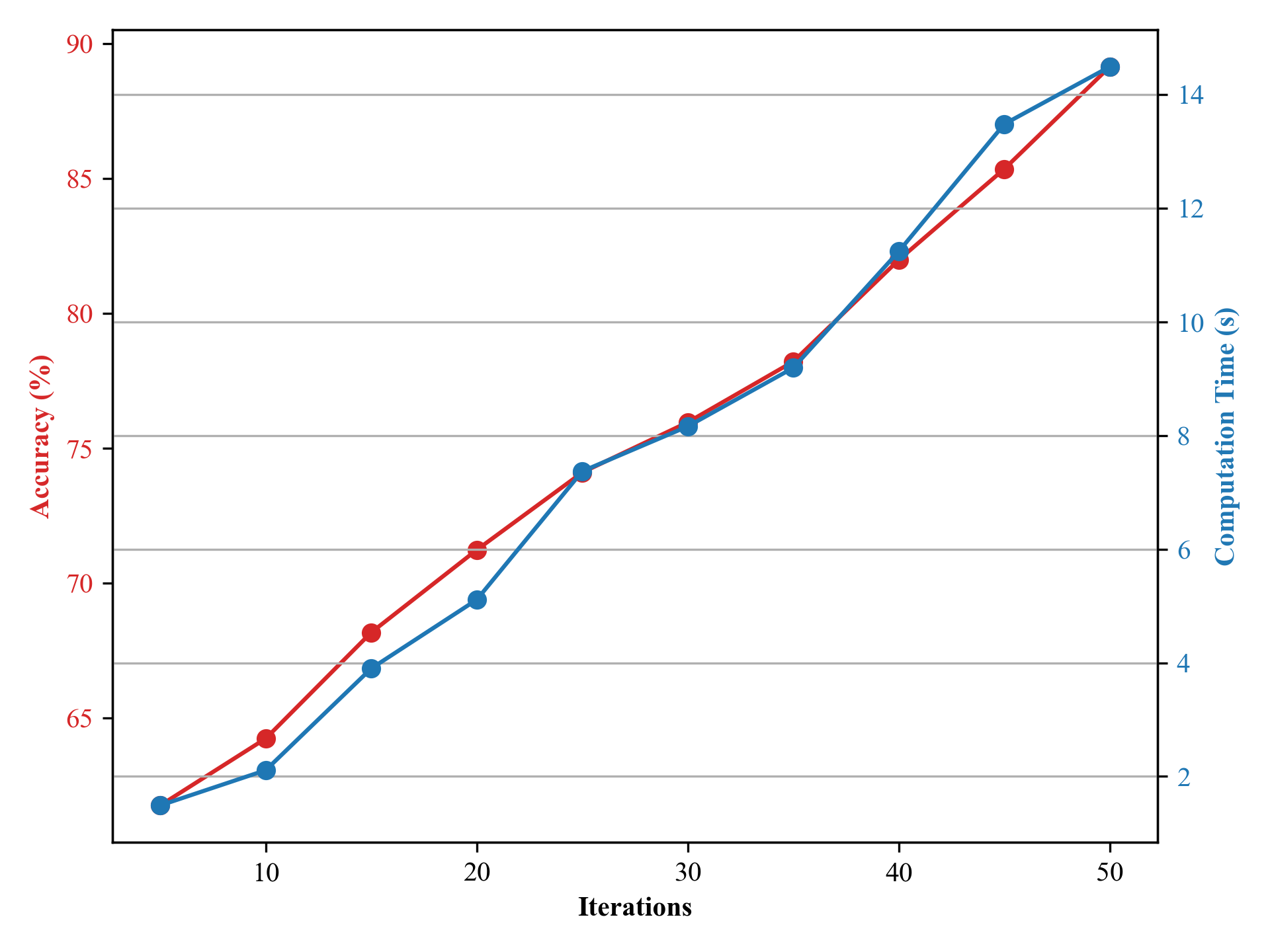

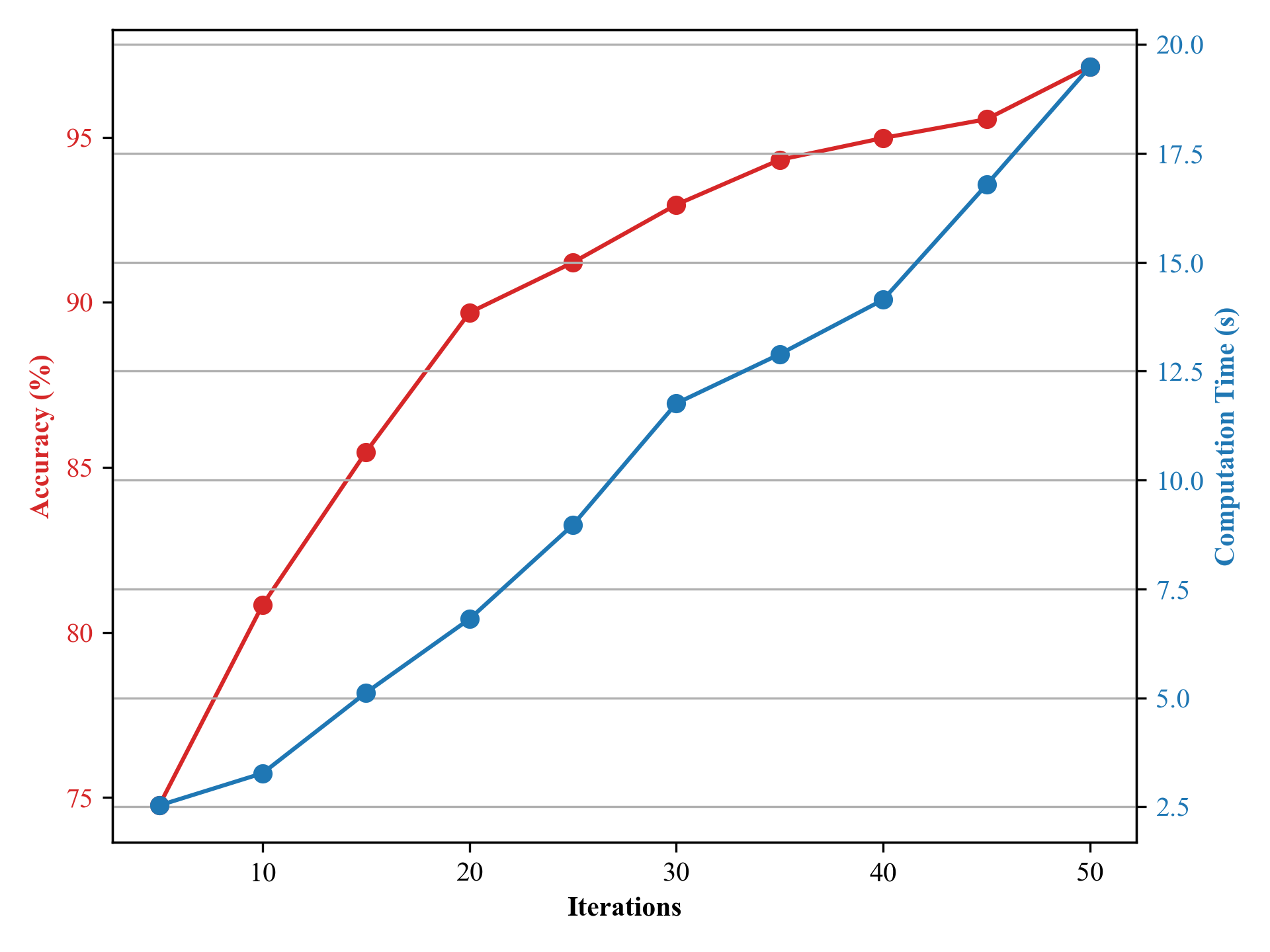


(a) (b)

Figure S8. Accuracy versus iterations versus time graphs for locomotion activities in
(a) the Extrasensory dataset and (b) the Opportunity dataset.


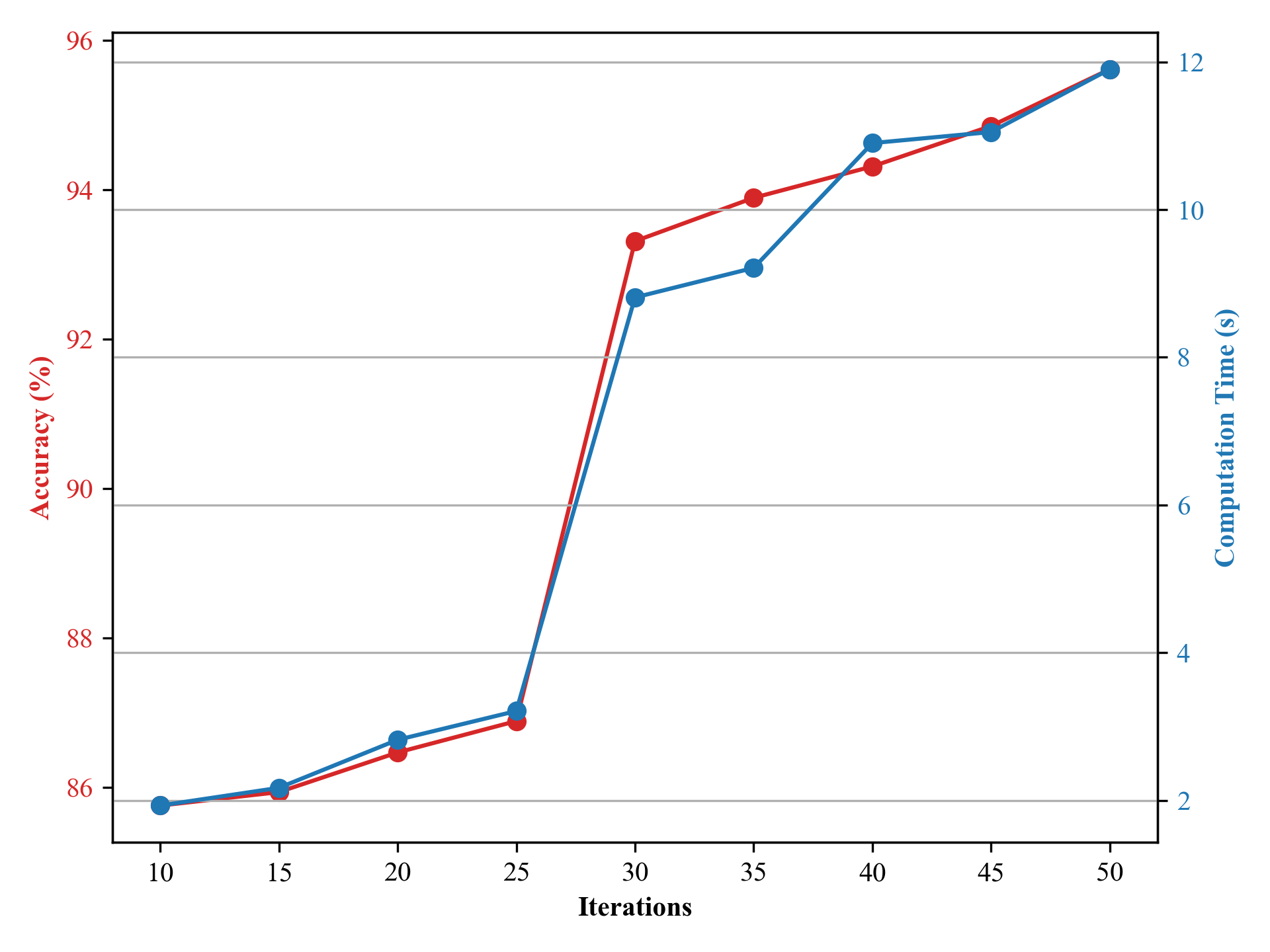


Figure S9. Accuracy versus iterations versus time graph for localization activities in the Extrasensory dataset.
